# Deconvolving Phylogenetic Distance Mixtures

**DOI:** 10.64898/2026.01.18.700179

**Published:** 2026-01-21

**Authors:** Shayesteh Arasti, Ali Osman Berk Şapcı, Eleonora Rachtman, Mohammed El-Kebir, Siavash Mirarab

**Affiliations:** 1 Department of Computer Science and Engineering, UC San Diego, CA 92093, USA; 2 Bioinformatics and Systems Biology Graduate Program, UC San Diego, CA 92093, USA; 3 Siebel School of Computing and Data Science, University of Illinois Urbana-Champaign, Urbana, IL 61801, USA; 4 Department of Electrical and Computer Engineering, UC San Diego, CA 92093, USA

**Keywords:** Mixture deconvolution, Phylogenetic distances, Metagenomics, Phylogenetic mixture analysis

## Abstract

Mixtures of multiple constituent organisms are sequenced in several widely used applications, including metagenomics and metabarcoding. Characterizing the elements of the sequence mixture and their abundance with respect to a reference set of known organisms has been the subject of intense research across several domains, including microbiome analyses, and methods must overcome two key challenges. First, the mixture constituents are related to each other through an evolutionary history, and hence, should not be considered independent entities. Second, sequence data is noisy, with each short read providing a limited signal. While existing approaches attempt to address these challenges, addressing both challenges simultaneously has proved challenging. For evolutionary dependencies, methods either define hierarchical clusters (e.g., taxonomies or operational taxonomic/genomic units) or use phylogenetic trees. For the second challenge, they either assemble reads into contigs, use statistical priors to summarize read placements, or attempt to analyze all reads jointly using k-mers. Despite this rich literature, a natural approach to simultaneously address both challenges has been underexplored: compute a distance from the mixture to all references, deconvolve those distances, and place the sample on multiple branches of a reference phylogeny with associated abundances. This multi-placement approach is a natural extension of the single-read phylogenetic placement used in practice. We argue that by placing the entire sample on multiple branches instead of placing reads individually, we can obtain a less noisy profile of the mixture. We formalize this approach as the phylogenetic distance deconvolution (PDD) problem, show some limits on the identifiability of PDDs, propose a slow exact algorithm, and an efficient heuristic greedy algorithm with local refinements. Benchmarking shows that these heuristics are effective and that our implementation of the PDD approach (called DecoDiPhy) can accurately deconvolve phylogenetic mixture distances while scaling quadratically. Applied to metagenomics, DecoDiPhy consolidates reads mapped to a large number of branches on a reference tree to a much smaller number of placements. The consolidated placements improve the accuracy of downstream tasks, such as sample differentiation and detection of differentially abundant taxa.

## Introduction

In many modern applications, DNA is sequenced not from a single organism but from a mixture. Examples abound and include sequencing of microbiome communities [1], heterogeneous cancer tumors [2], and true biological mixtures, such as admixtures [3–5] and recent hybrid species [6, 7]. A first step in analyzing these mixtures is characterizing their contents with respect to a reference dataset of known organisms, which faces several challenges. The constituents of a “query” mixture *and* the references are the product of an evolutionary process. Thus, query organisms are at some evolutionary distance from references (“novelty” hereafter), and while denser reference sets reduce novelty, in many applications, high levels of novelty remain, making simple sequence matching incapable of fully characterizing a sample [8–11]. Relatedly, the shared evolutionary history creates dependencies, and as a result, mixture identification should go beyond classifying into independent buckets. A second challenge is that mixture data most often consists of short reads with limited signal per read. The rich literature on mixture identification [12, 13] and particularly microbiome analysis [14, 15], which is our focus, has attempted to address these challenges in various ways. These attempts amount to ways to capture dependencies among species or among reads (Table S1).

Despite the rich literature, existing methods typically model either evolutionary dependencies or read dependencies, but not both. Most methods crudely model evolutionary histories by grouping reference genomes into predefined hierarchical taxonomic groups or data-derived [16–18] taxonomic/genomic operational units (OTUs/OGUs), and treat groups within a higher-rank category as independent. This provides limited modeling of dependencies, and the resulting taxonomic profiles have less resolution [19, 20] than using phylogenetic trees [21]. Methods for phylogenetic characterization also exist [22, 23], especially phylogenetic placement [24–29]. A rich toolkit uses phylogenetic profiles in downstream analyses such as comparing and clustering samples [30, 31], extracting phylogeny-aware features [32, 33] for supervised learning [34–37], and differential abundance across phenotypes [38, 39]. Separately, whether they use taxonomy [40–44] or phylogeny [24–29], most methods characterize individual reads and summarize results, often focusing on marker genes [45–47]. The summarization step can be as simple as averaging [48] or can further attempt to denoise the profile using prior expectations [49]. An alternative is to characterize the entire sample without separating reads [50, 51]. For example, k-mer sketching methods find which reference genomes seem present in a sample as a whole [52–54]. However, these methods often ignore evolutionary dependency, with limited modeling in some cases [55]. Thus, while the field has recognized both the importance of evolutionary dependencies and read dependencies, existing methods are limited in their ability to jointly address both challenges.

**Figure 1:**
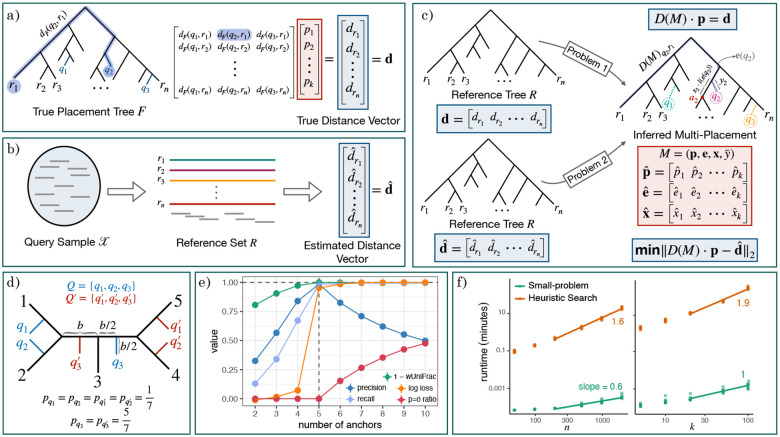
**a)** Illustration of the mixture distance d on the full tree F, and **b)** estimating $\overset{ˆ}{d}$ from sequence data (e.g., by mapping reads to references). **c)** PDDs infer multi-placements given the reference tree R and d or $\overset{ˆ}{d}$. **d)** Unidentifiable example. All $y_{q_{i}}=y_{q_{i}^{\prime }}=\frac{b}{2}$, and $x_{q_{i}}=x_{q_{i}^{\prime }}=\frac{1}{2}$ such that Q and $Q^{\prime }$ produce the same d. **e)** Exploration of the search algorithm on an example dataset with n=100 and true k=5. We show the proportion of placement edges that are correct (precision) and correct placements found (recall); 1–wUniFrac, a measure of accuracy incorporating p, x, and e; the normalized residue $\frac{{\text{max}}_{k}\left(\text{log}\;\ell_{k}\right)}{\text{log}\;\ell }$ (log loss); and proportion of abundances < 0.01 (p=0 ratio). Metrics improve for k≤5; using k>5 yields many p<0.01 values and small changes in loss. **f)** Empirical running time versus n and k, showing log-log plots; line slopes estimate the asymptotic power (excluding low n, k values).

We aim to address both challenges simultaneously by *i*) using a phylogeny instead of a taxonomy, while *ii*) placing the entire sample instead of placing one read at a time. To incorporate the phylogeny, we take advantage of the additive [56] metric space it defines; i.e., the pairwise path length between leaves. The rich literature on distance-based phylogenetics [57–59], including placement [60], has focused on input matrices where each row or column corresponds to a single leaf. The goal of this work is to go beyond this setting and enable distance-based phylogenetic placement where input distances are computed from mixtures. To that end, we pose the Phylogenetic Distance Deconvolution (PDD) problem where we are given a reference tree and a mixture query (Fig. 1a–c). The standard placement, adding the query as a single leaf [61], is insufficient because the input is a mixture. Instead, PDD seeks to insert the query into k edges of the tree. A distance is estimated from the entire mixture to each reference (a tree leaf), defined as the (weighted) average of distances from each constituent to that reference. PDD’s key novelty is to use the resulting mixture distance to place the sample on k branches. This effectively deconvolves the mixture into k separate distance vectors, each corresponding to one of the constituents, together with query abundances. PDDs, to our knowledge, have not been posed directly, though deconvolution without trees has been [50]. The closest attempt is MISA [62], which considered the special case of k=2 for a specific k-mer-based mixture distance. We argue that by considering the entire query set in one inference, PDDs provide an elegant formulation applicable to several applications, including metagenomics, on which we focus.

After presenting the PDD formulation, we start with the theoretical properties of PDDs, leaving some questions open. We then propose an efficient algorithm, implemented in a software tool called DecoDiPhy, by reformulating PDDs as a set of convex optimization problems and solving a small heuristically selected subset of those problems. We then evaluate the effectiveness of DecoDiPhy in a series of simulations and on real metagenomic data. Results show that by consolidating over-dispersed placements, DecoDiPhy is able to improve the accuracy of profiles and elucidate features associated with environments and clinical outcomes.

## Methods

### Notation and Problem Formulation

Let $T=\left(V_{T},E_{T}\right)$ be a tree with nodes $V_{T}$ and edges $E_{T}$. For a node $v\in V_{T}$, we let $C_{T}(v)$ denote the set of all the leaves in the subtree below v, and e(v) denote the incoming edge to v. For any edge $e\in E_{T}$, let $l_{T}(e)$ denote the length of the branch e in T (Fig. S1). We let $d_{T}(a,b)$ be the path length between two nodes a, $b\in V_{T}$. We often omit the subscript T when it is the reference tree R, defined below.

Consider a phylogenetic tree F on a leaf set labeled by ℛ∪𝒬 where 𝒬 represents a set of k query taxa and ℛ represents a set of n reference taxa, indexed by [k], [n]={1,…,n}, resp. We assume such a tree F exists, though we do not have access to it. Instead, we have access to a reference tree R, which we assume is F induced down to reference leaves ℛ (i.e., by pruning 𝒬 from F and suppressing degree-2 nodes). We refer to R as the *reference tree* and F as the *full tree*. We let m≤2n-2 denote $\left|V_{R}\right|$. We assume F is rooted arbitrarily and R keeps the same arbitrary rooting. Note that while we assume 𝒬∩ℛ=∅, branch lengths of F can be arbitrarily short; thus, if the query 𝒬 includes an organism q that is exceedingly similar to some reference r∈ℛ, we can include r and q in F with arbitrarily small branch length separating them. In addition to R, we have access to some data points (e.g., reads) 𝒳 gathered from a *phylogenetic mixed query* (𝒬, p), where each q∈𝒬 is associated with an abundance $p_{q}\geq 0$ such that $\sum_{q=1}^{k}p_{q}=1$ (i.e., $p\in \Delta^{k-1}$ where Δ denotes a (k-1)-simplex). Each x∈𝒳 belongs to an (unknown) constituent q∈𝒬, and the proportion of 𝒳 belonging to q follows $p_{q}$ (i.e., 𝒳 can be modeled as a random draw according to p). Let the query-reference distance matrix $D(F)\in R^{n\times k}$ be defined by $D(F{)}_{r,q}=d_{F}(r,q)$. We define the *(true) mixture distance* vector $d\in R^{n}$ as d=D(F)⋅p (Fig. 1a). If we do not have access to d, we assume we can estimate $\overset{ˆ}{d}$ from 𝒳 such that (ideally) $E[\overset{ˆ}{d}]=d$. The goal is to recover F and p from R and either d, or $\overset{ˆ}{d}$ estimated from 𝒳. Note that in this general form, F can have splits (i.e., unrooted edges) made entirely of subsets of 𝒬. For a query q, its *true placement edge* on R is an $e\in E_{R}$ such that if q is inserted as a new child by splitting e, the resulting tree remains compatible with F (see [63] for the definition of compatibility).

We begin with the case where the true d is given to study the identifiability of the problem.

**Problem 1** (Phylogenetic Distance Deconvolution (PDD)). *Given a reference tree* R *on* ℛ=[n]*, a mixture distance vector* $d\in R^{n}$*, and* k*, find a full tree* F *with* n+k *leaves that is compatible with (i.e., induces)* R *and includes* k *additional query leaves and their proportions* $p\in \Delta^{k-1}$ *such that*

(1) d=D(F)⋅p

Even with error-free input d, for F to be recoverable, we need identifiability; that is, if (F, p) satisfies Eq. (1), there should exist no other ($F^{\prime }$, $p^{\prime }$) that also satisfies it. However, PDD is only partially identifiable.

**Claim 1.** *If two queries* q *and* $q^{\prime }$ *share the same placement edge on* R*, both queries can be replaced with a single query with abundance* $p_{q}+p_{q^{\prime }}$ *without changing the distance mixture* d.

**Claim 2.** *Problem 1 is not identifiable if* $x_{q}=0$ *or* $x_{q}=1$ *are allowed. Thus, we only allow* $x\in (0,1{)}^{k}$*. Also, any two vectors* y *and* $y^{\prime }$ *with* ${y^{\prime }}^{⊤}p=y^{⊤}p$ *lead to the same distance mixture* d *in Problem 1. Thus, we can only identify the mean terminal branch length* $\bar{y}={\text{y}}^{⊤}\text{p}$ *and not the individual values of* y.

**Claim 3.** *Even for fixed (true) placement edges, the relative positions* $x_{i}$ *and proportions* $p_{i}$ *are unidentifiable under some corner conditions for queries placed on adjacent edges*.

All claims are proved in Section SB.3. Because of these negative results, we need to disallow two queries being placed on the same edge of R. Essentially, we can identify the total p subtending from each branch of R, but cannot deconvolve the relationship per branch. More formally:

**Assumption 1.** *No two queries share a placement edge. Thus, a solution to Problem 1 can be expressed as* k *distinct placements on* R *represented by vectors* e,x,p *indexed by* [k] *and* $\overset{‾}{y}$; $e_{q}\in E_{R}$ *indicates the placement edge,* $0<x_{q}<1$ *is the relative position of* q *defined as the distance from the subtending point of* q *to head of* $e_{q}$ *divided by* $l\left(e_{q}\right)$*, and* $p_{q}$ *is its abundance (*Fig. 1c*). We only seek* $\overset{‾}{y}=y^{⊤}p$ *and not* y.

Even with Assumption 1, the problem is still *not always* identifiable, as shown by a counter-intuitive counterexample, showing two very different query sets with identical d (Fig. 1d). Despite this lack of strict identifiability, we suspect (with no proof) that conditions that break identifiability require extreme cases of symmetry similar to Figure 1d that are not expected on real data. We empirically explored identifiability by randomly sampling PDD instances and solving them exactly and did not find any unidentifiable cases, indicating those may be rare. We leave a full characterization of identifiability to future work.

Problem 1 can be expressed linear algebraically using auxiliary variables. Let $A:{\left(V_{R}\right)}^{k}\to \{0,1{\}}^{m\times k}$ be a binary representation of e defined by $A(e{)}_{v,q}=1$ iff query q is placed above node v (i.e., $(v)=e_{q}$). Note that $\forall q:\sum_{e\in E}A_{e,q}=1$ and $\forall e:\sum_{q}A_{e,q}\leq 1$, where the second constraint follows from Assumption 1. We let the *anchor node* $a_{q}\in V_{R}$ of q be the tail of its true placement edge. Let $w\in (0,1{)}^{k}$ be w=x∘p where ∘ is element-wise product and note that $w_{q}\leq p_{q}$. Let $C\in \{-1,1{\}}^{n\times m}$, where $C_{r,v}=1$ iff r∈C(v) (r descends from v) and $C_{r,e}=-1$ otherwise; $L\in R^{m\times m}=\text{diag}\left(l\left(e\left(v_{1}\right)\right),\ldots ,l\left(e\left(v_{m}\right)\right)\right)$ where $V_{R}=\left\{v_{1}\ldots v_{m}\right\}$. Finally, $D\in R^{n\times m}$ is a distance matrix equivalent to tree R where $D_{r,v}=d_{R}(r,v)$ for r∈ℛ, $v\in V_{R}$.

**Claim 4.** *Under conditions of Assumption 1, we can rewrite* Eq. (1) *as*

(2) $$d=D\cdot A\left(e\right)\cdot p+C\cdot L\cdot A\left(e\right)\cdot w+\overset{‾}{y}\cdot 1_{n}\;\text{s.t.}\;\forall q:w_{q}\leq p_{q}.$$

We can rewrite Problem 1 using Eq. (2), noting that D, C, L are given and A(e), w, p and $\overset{‾}{y}$ are unknown variables. e gives placement edges. Attachment positions on each can be recovered as xq=wqpq, provided $p_{q}>0$. While we can only recover $\overset{‾}{y}$ (Claim 2), in practice, we can use heuristics such as setting $y_{q}=\overset{‾}{y}$ or setting $y_{q}$ proportionally to mean height of $C\left(a_{q}\right)$ to $a_{q}$ such that they sum up to $k\overset{‾}{y}$ across all queries.

On real data, we do not have access to true mixture distances. Instead, we can *estimate* mixture distances from sequence mixtures (𝒳). We revisit methods for computing $\overset{ˆ}{d}$ later, treating it as a separate problem from PDD. In theory, we need a statistically consistent estimator of additive tree distances from 𝒳. However, due to noise and problem structures like additivity of the tree metric, erroneous data may admit *no* solution to Problem 1. To accommodate error, we form an optimization problem. We need a metric to quantify the similarity of two distance vectors. A natural choice is $\ell_{2}$, which is easy to optimize. We can now define:

**Problem 2** (PDD optimization). *Given a reference tree* R *and an estimated mixture distance vector* $\overset{ˆ}{d}\in R^{n}$*, infer a multiplacement* (p, e, w, $\overset{‾}{y}$*) that adds* k *queries to* R *by minimizing:*

(3) $${\left\|\overset{ˆ}{d}-\left(D\cdot A(e)\cdot p+C\cdot L\cdot A(e)\cdot w+\overset{‾}{y}\cdot 1_{n}\right)\right\|}_{2}^{2}$$

$$\text{s.t.}\;0<w<p,0<\overset{‾}{y},\sum p=1,\forall q:\sum_{e\in E}A_{e,q}(e)=1,\forall e:\sum_{q}A_{e,q}(e)\leq 1.$$

Among variables of Problem 2, A(e) and k are combinatorial in nature and hard to optimize directly.

### DecoDiPhy Algorithm

#### Exact solution.

If we fix A in Problem 2, we get a “small-problem” of minimizing (p, w, $\overset{‾}{y}$) in Eq. (3), which is a simple quadratic objective function due to the linearity of Eq. (2). Since the objective function is convex, the small-problem has a unique minimization cost; however, the objective function is not strictly convex and can have multiple minimizers. Nevertheless, convexity of Eq. (3) allows an exact algorithm:

**Definition 1** (Exhaustive search). *For every possible* k*, for every possible matrix* A*, optimize the quadratic objective function of* Eq. (3) *using gradient descent; choose* (k, A, p, w, $\overset{‾}{y}$) *that minimizes objective value*.

This exhaustive algorithm is not practical. Even if we limit ourselves to a constant range 2≤k≤K, we need to explore ∑k=2Kmk=OnK possible A matrices. Ignoring the time for each optimization, we will need $O\left(n^{K}\right)$ steps, which is practical only for small K (e.g., K=3), even for moderately sized trees. A further complication is that on noisy data, as k increases, the objective function tends to decrease. Thus, we cannot simply rely on the optimization score to choose the right k; instead, we need a heuristic to select k.

#### Heuristic search:

We developed a heuristic search algorithm (Algorithm 1) adopting a greedy search with local refinements. Briefly, we add one placement at a time, starting from k=1 (Line 15). For each k, we start from the optimal solution found for k-1, and test all possible placement edges (Line 3) for the kth query (Line 6), solving the small-problem (i.e., quadratic optimization) to minimize Eq. (3) for each edge visited and selecting the placement with the minimum residue. We then revisit all prior k-1 queries and test if their placement can improve (Line 18), fixing all other queries; we accept any move that reduces the residue. When revisiting prior nodes, we search a constant number of edges (Line 5), limiting the search to edges that are up to ρ edges away from the best previous placement (i.e., limited radius). We stop for any k only when no improvement is possible. We use Algorithm S1 to handle boundary cases of Claim 2. For large backbones ($n>10^{3}$), we use divide-and-conquer (Section SB.2) to solve PDDs on several subtrees.

#### Selecting k.

Let us examine an example dataset (Fig. 1e). With k=2, some placements are correct and others are phylogenetically close. This observation motivated our semi-greedy heuristic. All metrics improve as k approaches its true value. When we reach the true k, the residue drops dramatically; however, this pattern becomes blurred with noisy data (Fig. S2), so we do not use it by default. Instead, note that for i>k, the extra placements tend to lead to many placements with $p_{q}\approx 0$. Thus, we stop exploring higher k when at least one $p_{q}\approx 0$ (Line 23), defined as pq<103𝒳, allowing lower abundance as coverage increases.

#### Asymptotic time.

D and C⋅L can be easily pre-computed once in $O\left(n^{2}\right)$ time, and multiplying with A(e) is simple column selection. Because the greedy refinement is allowed to continue moving queries until no improvement is observed, the asymptotic running time is not strictly bounded. For exposition, let us bound the number of rounds of optimization in Line 18 by C and bound k (Line 15) by K; then, we will need Θ(K) outer loops (Line 15), each with O(C) rounds of improvement, each with O(K) queries moved across a constant number of edges, and the $k^{\text{th}}$ query moved across Θ(n) edges. Thus, in total, we run the small-problem $O\left(C\left(K^{2}+Kn\right)\right)=O(CKn)$ times. In our experiments, C is nearly constant versus n and k (Fig. S3), resulting in O(Kn) runs of small-problem, compared to $O\left(n^{K}\right)$ for the exact algorithm. We implemented the small-problem using the CVXPY [64] package for convex optimization, using the OSQP [65] optimizer, which is a first-order iterative method (ADMM). Empirical tests show that the small problem with Θ(K) variables and Θ(n) equations scales with $O\left(K\cdot n^{0.6}\right)$ (Fig. 1f). Sublinear dependency on n may be due to redundancies and/or sparsity in matrices and the highly optimized implementation within CVXPY. Considering the total number of rounds, the total empirical running time increases with $O\left(K^{2}n^{1.6}\right)$ (Fig. 1f).

### Estimating distance vector $\overset{ˆ}{d}$

While DecoDiPhy can take any $\overset{ˆ}{d}$ as input, we explore two methods. *i*) Sequence-based $\overset{ˆ}{d}$: Assume genomes evolve down F under the simple Jukes-Cantor (JC) [66] model. Then, let t(h)=-34log1-43h and let $h_{x,r}$ be the Hamming distance between an error-free read x from query q and a reference genome r if x is aligned correctly to r. Then, $t\left(h_{x,r}\right)$ is the ML estimator of $d_{F}(r,q)$ [66]; thus, the mean of $t\left(h_{x,r}\right)$ over x∈𝒳 converges to mixture distance $d_{r}$, giving us a valid estimator $\overset{ˆ}{d}$. For computing distances for genome-wide reads, we can use read alignment or the more scalable alignment-free method, krepp [29]. *ii*) Placement-based $\overset{ˆ}{d}$: We also use PDDs to consolidate outputs of per-read placement methods. We use an existing method to place each read x∈𝒳 on R and let $\overset{ˆ}{F}$ be the resulting tree, creating polytomies for multiple placements on the same branch. We simply use the mean distance dˆr=1|𝒳|∑xdFˆ(r,x). While individual placements may be wrong, if $E\left[d_{\overset{ˆ}{F}}(r,x)\right]\approx d_{F}(r,q)$ (expectation is over all x that belong to q), then $\overset{ˆ}{d}\approx d$. We use krepp [29] to obtain per-read placements, setting x=12, y to the height of the sister clade. krepp can be configured to output one or multiple placements per read. We explore both modes, and for multi-placements, we take the average distance per read.

**Table:** 

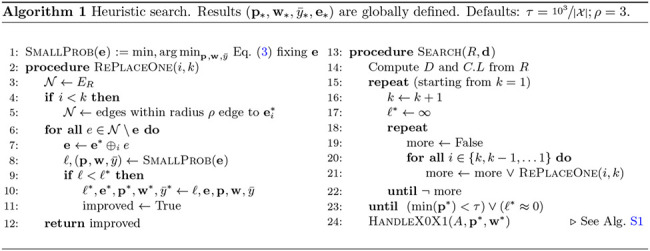

### Experiment Design

#### E1: Heuristic search.

We first evaluate the effectiveness of our search heuristic using 12 biological trees with n≤363 (Table S2). We compare Alg. 1 to the exact version (Definition 1), feasible for k∈{2,3} and n<120, and a version of Alg. 1 with true k given or with ρ set to ∞ to perform a more thorough search. We randomly selected k∈{2,3,5,7,10} query species (only keeping k≤n3 cases) and assigned a random abundance vector $p\in \Delta^{k-1}$ (Section SB.4.2). The query taxa were pruned to obtain R (10 replicates). We tested both true distances d and a noisy estimate $\overset{ˆ}{d}$, meant to reflect errors in placing individual reads on R. The procedure (Section SB.4.2) essentially divides queries into 10^5^ pieces and moves each piece around on the tree stochastically, computing $\overset{ˆ}{d}$ from the noisy placements. We use two settings with pieces moving by an average of 0.6 or 1.56 edges. Since no other method is specifically designed for PDDs, we compared DecoDiPhy to three baseline algorithms, all given true k: reverse-search (apply the small PDD on all edges, remove low $p_{q}$ branches, and recurse), k-nearest leaves by distance, and a greedy k-nearest leaves with feedback (removing contribution of already selected leaves before selecting the next leaf); see Section SB.4.2.

#### E2: Impact of distance computation.

We next tested the impact of *how* the input mixture distance vector $\overset{ˆ}{d}$ is computed in a more realistic setting, focusing on two relatively small subtrees with n=34 and 111 (see Section SB.4.3). For each subtree, we simulated five sets of query taxa for k∈{3,5,10} together with randomly drawn abundances. We simulated 10^5^ short 150bp reads with errors from query genomes using ART [67] and ran DecoDiPhy with both true and estimated distances. For distance computation, we used both sequence-based and distance-based methods described earlier, implemented using krepp [68], which is a k-mer-based method that estimates read-to-reference (Hamming) distances as well as either a single placement branch or multiple equally likely placement branches (multi-placements) for each read.

#### E3: Simulated metagenomes.

We simulated realistic metagenomes using leave-out experiments on the WoL-2 [69] reference tree with 15,953 leaves to compare DecoDiPhy to existing metagenomic profiling methods. Zhu *et al*. [17] have mapped 210 HMI (Human Microbiome Initiative) samples across 7 human body sites to WoL2 genomes using Woltka [17], producing a vector $p^{\left(i\right)}$ of normalized OGU counts for each sample i. To mimic these microbial communities, we randomly selected 50 samples, and for each, drew a count for each genome from $\text{Multinomial}\left(N;p^{\left(i\right)}\right)$ and normalized these counts to obtain the ground-truth abundances p. For each sample, we used $N\in \left\{10^{2},10^{3},10^{4}\right\}$ to control true k (low, med, high; Fig. S4a), and left out either some or all queries from R to create either low or high novelty levels (Section SB.4.4). For each case, we simulated 10^6^ short reads with errors using ART [67] and analyzed these using krepp (multi-placements) [29] with and without DecoDiPhy. Since no other method performs phylogenetic placement on such a scale, we compared with Wotlka, which maps reads to genomes using bowtie2 [70], and sylph [54], which assigns the entire sample to genomes (Section SB.4.5), treating results as placing on leaf branches of R. We were able to only test methods that scale to WoL-v2 reference set, assign abundances to genomes as opposed to taxonomic units (incongruent with R), and can easily leave out chosen “query” genomes.

#### Evaluation.

We used four widely used metrics from ecology to compare true and estimated multi-placements. *Jaccard index* (intersection/union) measures the similarity of placement edges alone, ignoring p, x, y, and R. *Bray-Curtis (BC)* [71] is similar to $\ell_{2}$ norm; it incorporates abundances p but ignores the tree. *UniFrac* [30] and *weighted UniFrac (wUniFrac)* [72] take into account R (unshared spanned branch length over total spanned branch length); UniFrac ignores p while wUniFrac considers it. To compute (w)UniFrac, we place true and estimated queries on R but set all ys (but not xs) to 0 for true and estimated placements because the alternative methods cannot compute y and our method can compute only $\overset{‾}{y}$.

### Biological Experiments

We studied two metagenomic datasets representing contrasting scenarios: an IBD (Inflammatory Bowel Disease) dataset with 220 human gut samples from diseased subjects and controls [73], and a subset of EMP (Earth Microbiome Project) dataset with 210 samples collected from free-living communities [74] (Section SB.4.6). We analyzed both datasets using the 15,953-taxon WoL2 [69] reference set. Compared to EMP, which originates from poorly characterized environments, the IBD samples are expected to have lower novelty relative to WoL2. We compared DecoDiPhy, with krepp multiplacements as input, to six methods, including those from E3 plus sourmash [52], Kraken-II [41], and Bracken [49], which we could not include in simulations because query leave-out is expensive for these methods. To evaluate accuracy, we examined the separation between diseased and control samples for IBD and between saline and non-saline free-living samples in EMP. For each dataset, samples were split into training (70%) and testing (30%) sets. Differentially abundant (DA) features were identified on the training set using ANCOM-BC [75], implemented in QIIME 2, given class labels, and selecting those with a q-value < 0.005. Note that each feature is an internal or terminal branch on the phylogeny for krepp and DecoDiPhy, a genome or terminal branch on the phylogeny for sylph, sourmash, and Woltka, and taxonomic label for Kraken and Bracken. Pairwise sample distances on the test set were computed using either all features or the DA features based on BC and (w)UniFrac, using the taxonomic tree (all branch lengths set to 1) for Kraken and Bracken. We used the PERMANOVA test of whether the separation of labels is significantly better than random. See Section SB.4.6 for commands used.

## Results

### Simulation studies

#### E1:

**Figure 2:**
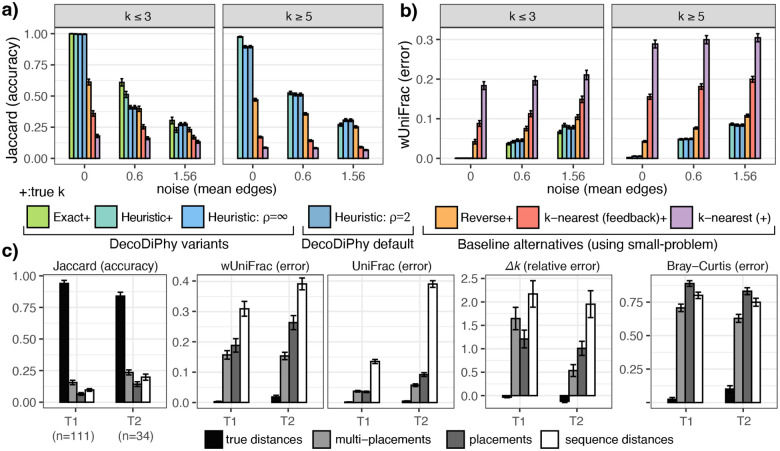
**a,b)** E1: Impact of search algorithm. Bars show the mean and standard error (120 points for noise=0 and 240 points for other noise levels) for four variants of DecoDiPhy and three baseline methods, which still use our small-problem internally, on 12 biological trees. Note that Exact variant could be run only for k<4. x–axis: noise in input distance, measured as the mean number of branches by which each placement is moved. + indicates a method is given the true k. For full results, separating all k values and showing other metrics, see Fig. S5. **c)** E2: Impact of distance calculation method, comparing true distances, distances from a placement method, or from sequences, on two small subtrees. Metrics consider different aspects: Jaccard only placement edges, Bray-Curtis only placements and abundance, UniFrac only placements and branch lengths, wUniFrac: placement, branch length, and abundance.

Given true distances, DecoDiPhy finds the true placements in all cases with the exhaustive search (Definition 1), showing that the situations where PDDs lack identifiability are rare (Fig. 2a). Our heuristic search (Alg. 1) also achieves close to perfect accuracy given the true k and d, demonstrating its effectiveness. The heuristic to select the cardinality k occasionally underestimates k (Fig. S5), leading to a 10% drop in Jaccard accuracy. Nevertheless, we see low wUniFrac errors (Fig. 2b), which incorporate R and p, showing that the missing placements are among low abundances and obtained placements are phylogenetically close to true placements. Not limiting the radius of refinements (setting ρ=∞ in Line 5) does not improve accuracy, but increases the runtime by O(n). None of the three alternative methods was nearly as accurate as DecoDiPhy. The nearest k neighbor has very high error, which is reduced by the feedback mechanism but does not become competitive with DecoDiPhy. The closest accuracy to DecoDiPhy is the reverse search using our small-problem. This is far better than nearest neighbors but not as accurate as DecoDiPhy. When noise is added to the input ($\overset{ˆ}{d}$), Jaccard accuracy drops for all methods, and other measures of error increase (Fig. S5). However, wUniFrac (Fig. 2b) does not increase nearly as much as Jaccard decreases (Fig. 2a), showing that errors tend to be among closely related and low abundance placements. Judged by wUniFrac, there is very little difference between the exact and heuristic versions of DecoDiPhy given noisy input.

#### E2:

When we infer distances from simulated reads, we observe a substantial drop in Jaccard accuracy but a more modest degradation by the tree-aware wUniFrac metric (Fig. 2c). There is a marked difference between methods for computing $\overset{ˆ}{d}$, with placement methods outperforming sequence-based, and multi-placements (which model uncertainty) working better than single placements. Placement-based $\overset{ˆ}{d}$ generally correlates with d better than sequence-based ones (Fig. S6), though both measures have substantial inaccuracies (Pearson correlation over all replicates: 0.89 and 0.69 for multi-placements and distances, resp.) Our heuristics to detect k tend to overestimate it with the noisy input, especially with lower k (Fig. S7).

#### E3:

**Figure 3:**
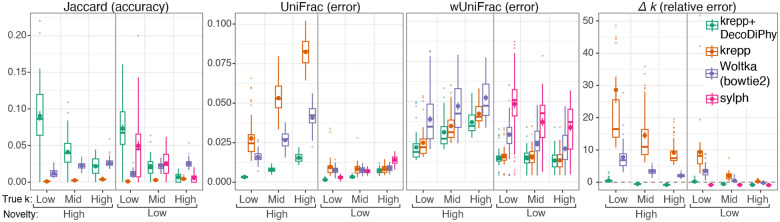
E3: realistic metagenome simulation. For six model conditions (two novelty levels × three true ks), 50 replicates each, we compare independent read placement (krepp), DecoDiPhy run on krepp, single read mapping (Woltka), and joint sample mapping (sylph). On high novelty, sylph found no matches. UniFrac and k relative error are limited only to species with abundance at least 10^−4^ while other metrics consider all placements. See also Fig. S4.

Our large-scale simulations seeking to emulate real data showed that compared to its input (krepp), DecoDiPhy dramatically reduces the placement error for novel and low complexity (i.e., low k) conditions with smaller improvements for low novelty+high complexity input (Fig. 3). DecoDiPhy is also far more accurate than read mapping using Woltka(bowtie2). On a tree with 16k leaves, Jaccard accuracy is unsurprisingly low, as many placements can move to nearby near-identical genomes (this same phenomenon makes genome-level Bray-Curtis distances less useful with 32k units). Nevertheless, DecoDiPhy can find the exact correct placement for up to 15% of queries for both novelty levels, followed by sylph in the low novelty case.

The accuracy of all methods depended on novelty. With higher novelty, sylph, which was quite accurate on lower novelty data, was not able to match any queries. DecoDiPhy had the lowest degradation in accuracy moving from low novelty to high. The most dramatic improvements with DecoDiPhy are observed for UniFrac for the high novelty condition, where error is reduced by 9× and 4× compared to krepp and Woltka, respectively (improvements are even larger if we don’t filter low abundances; see Fig. S8). The improvements in wUniFrac are smaller than UniFrac, consistent with the notion that DecoDiPhy consolidates krepp’s placements, reducing noisy low-abundant placements. The complexity of the sample also mattered, though less than novelty (in our setup the two are not fully independent; Fig. S4). UniFrac error increases with complexity, but wUniFrac only increases in high novelty conditions. The detected number of queries was too high by 5–30× in most conditions for krepp and Woltka, whereas sylph detected fewer queries than present. DecoDiPhy detected slightly too many for low complexity and too few for high complexity cases.

### Biological metagenomic analyses (IBD and EMP)

**Figure 4:**
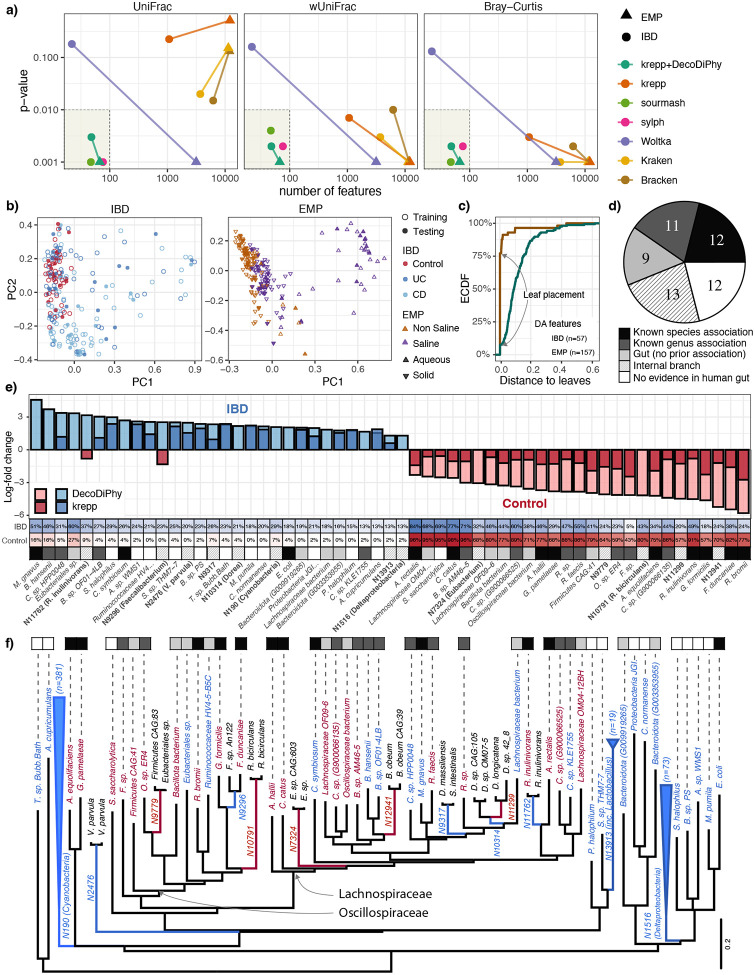
**a)** For both IBD and EMP datasets, we show the number of tree edges (i.e., features) marked as differentially abundant (DA) across categories (healthy/diseased for IBD, saline/non-saline for EMP), versus the p-value testing separation between the categories according to each distance metric using the PERMANOVA test. For Kraken and Bracken we use taxonomy with all branches set to 1 for (w)UniFrac. DA fails for sourmash and sylph on EMP due to few placements recovered. Shaded: desired regime with few features that separate categories. **b)** Principle coordinates of wUniFrac for DecoDiPhy on both datasets, showing separation between categories used to find DAs; for EMP, PC1 separates solid versus aqueous, though DA features were selected for saline/non-saline, not this categorization. **c)** Distribution of the distance from each DA feature to leaves of the tree (mean); most IBD features are leaves while EMP is internal nodes. **d,e,f)** DA features on IBD data, together with their log-fold change (LFC) in abundance; the tree shows these features. LFC of krepp is shown if available. For all DA leaves, we show whether they are supported by the literature (see also Table S3).

Without access to ground truth, we ask which methods can find a relatively small number of differentially abundant (DA) placements (i.e., features) on a training set that significantly distinguish labels on the separate testing samples (Fig. 4a). On the *IBD* dataset, DecoDiPhy, sourmash, and sylph found 48, 47, and 76 DA features, resp., and yet achieved statistically significant separation (p-value < 0.01) for UniFrac, wUniFrac, and Bray-Curtis (BC) metrics. Woltka did not achieve significant separation, while krepp, Kraken, and Bracken did so only with abundance-aware metrics using at least 1000 DA features, reducing the interpretability. Using the DA features, healthy samples show less variation than IBD samples; we see no strong separation between the two subtypes of IBD (UC/CD) or training and testing data (Fig. 4b).

On the EMP dataset–more diverse and less well represented than IBD–sourmash and sylph found very few matches, which prevented reliable DA analysis (Fig. S9). Their inability to match novel EMP samples is consistent with their reduced performance in (E3) simulations with higher novelty. Remaining methods significantly separated saline from non-saline, except for krepp+UniFrac (Fig. 4a). However, alternative methods identified thousands of DA features (Fig. S9), limiting their interpretability. DecoDiPhy achieved significant separation using only 67 DA features (60 are internal nodes). Although DA features were identified with respect to the salinity, the first principal coordinate also separates solid and aqueous samples (Fig. 4b).

Examining all placements (i.e., not just DA ones), DecoDiPhy reduced krepp placements five to six folds, with better separation according to UniFrac and comparable according to other metrics (Fig. S9b). Both sourmash and sylph had three to five times fewer features than DecoDiPhy and great separation on IBD; on EMP, sourmash had good separation while sylph failed to find any feature for around 13 of samples.

Combining training and testing, DecoDiPhy yielded 57 DA features for the IBD dataset and 157 for the EMP. Examining the phylogenetic depth of DA placements (the average path length from descending leaves to the edge) showed the impact of novelty. In the IBD dataset, 77% of DA features corresponded to terminal branches (genomes) compared to 7% for the EMP dataset (Fig. 4c) where internal placement was essential.

For the IBD dataset, we searched the literature to assess the validity of the DA features (Fig. 4de). Most identified features belong to two well-studied families *Lachnospiraceae* and *Oscillospiraceae* implicated in gut health and IBD [76–79] (Fig. 4f). Among 44 genome-level features, 12 species have prior evidence of association with IBD in the same direction we observed (Fig. 4d; Table S3), eight depleted (*Ruminococcus bromii, Faecalibacterium duncaniae, Gemmiger formicilis, Roseburia inulinivorans, Adlercreutzia equolifaciens, Gordonibacter pamelaeae, Anaerobutyricum hallii, Coprococcus catus*, and *Agathobacter rectalis*) and three enriched (*Mediterraneibacter gnavus, Clostridium symbiosum*, and *Escherichia coli*). Notably, 7 and 8 of these 12 species were also detected by sourmash and sylph, resp. (Fig. S10), while others were missing, and three of the 12 (Fig. 4e) were not among 1818 features found by krepp (i.e., the input to DecoDiPhy). Among the 44, we also detected 11 unclassified species from genera with well-documented roles in IBD. All of these genomes were also detected by sourmash and/or sylph, and the direction of association and the log-fold changes were consistent across methods (Fig. S10). Seven species (from genera *Faecalibacterium*, *Oscillibacter*, *Ruminococcus*, *Clostridium*, and *Blautia*) were identified as depleted in IBD, consistent with the literature [80–83], while four (from genera *Coprococcus*, *Blautia*, and *Clostridium*) were enriched. For *Blautia* and *Clostridium*, our bidirectional associations are expected: both genera contain taxa reported to be reduced in some IBD cohorts and expanded in others, reflecting their mixture of anti- and pro-inflammatory members [84–89]. While *Coprococcus* is generally depleted in IBD [84, 90], the particular species (Coprococcus sp. HPP0048) identified has been reported to *increase* in the related postinfection irritable bowel syndrome (PI-IBS) [91]. Additionally, we identified 9 genomes from groups known to appear in the human gut, but without prior evidence from the literature for IBD association. We identified 12 enriched genomes (including two archaea) with no prior evidence of gut association. These all appear in krepp’s DA features, often with higher log-fold changes (Fig. 4e). Their estimated distances to assigned reference genomes (proxy for y) are consistently higher than features found in literature, suggesting that these 12 features may reflect references missing from the tree, leading to placements on phylogenetically related branches of the tree (Fig. S11).

DecoDiPhy also identified three internal edges with subtrees of at least 10 genomes enriched in IBD (Fig. 4f). One of these (N13913) is a long branch (length > 0.08) that includes a tight cluster (diameter < 0.06) of *Lactobacillus* genomes. Although *Lactobacillus* species are often considered beneficial commensals or probiotics [92], several studies have reported increased abundance of certain *Lactobacillus* strains in IBD cohorts [79, 93]. A second example (N1516) corresponds to the class *Deltaproteobacteria*, which includes several sulfate-reducing bacteria (e.g., *Desulfovibrio, Desulfomicrobium*) [94]. These taxa have been repeatedly associated with IBD and mucosal inflammation [95, 96]. The third one (N190) corresponds to the least common ancestor of the phylum *Cyanobacteria*, with only limited evidence connecting it to IBD [81, 90].

Finally, presence/absence across the two groups reveals an interesting pattern (Fig. 4e). Features enriched in healthy controls tended to be present in a *larger fraction* of both control and IBD samples (although at low frequency in IBDs), whereas features enriched in IBD tended to be *less prevalent overall and* were often missing from controls. This pattern aligns with established models of gut dysbiosis in IBD, in which disease can arise through both *loss-of-function* (loss of widespread beneficial taxa) and *gain-of-function dysbiosis* (the expansion of less common, potentially pathogenic or pro-inflammatory taxa) [97, 98].

On the EMP dataset, DecoDiPhy detected 157 DA features (11 terminal and 146 internal branches), of which 108 were enriched in non-saline samples and 49 in saline samples. This pattern is consistent with prior observations that microbial diversity generally decreases with increasing salinity [99, 100]. Several higher-level clades—most notably the phyla *Gemmatimonadota, Bacteroidota*, and *Pseudomonadota*—were enriched in saline samples, in agreement with previous reports of salt-tolerant or halophilic lineages within these groups [101–103]. We also observed that DA features enriched in saline samples tended to correspond to deeper, higher-rank branches (mean height = 0.17), whereas features enriched in non-saline samples were more often from lower taxonomic levels (mean height = 0.10; Table S4). This pattern reflects the well-established trend that saline environments are dominated by a select number of highly diverse (salt-adapted) clades, whereas non-saline environments support greater diversity at finer taxonomic scales [104–106].

## Discussion

We proposed the phylogenetic distance deconvolution (PDD) approach for the widely studied mixture decomposition problem. PDD combines two features, each present in existing methods but not previously together: allowing phylogenetic placements and enabling joint characterization of reads. Compared to taxonomic assignment, phylogenetic placement provides finer resolution [17, 107], and unlike methods that assign reads only to leaves [17, 23, 54], PDD allows insertion on internal edges, covering half the tree. PDD is expected to outperform existing phylogenetic methods by reducing over-dispersion of placements (producing more interpretable results). We expect it to outperform methods that jointly model reads (e.g., sylph, sourmash, Quikr) when input is phylogenetically novel relative to the reference.

Our simulations and empirical analyses support these expectations. When novelty is low (e.g., IBD), sourmash and sylph perform well and match or outperform DecoDiPhy. Additionally, sylph is extremely scalable, taking less than a minute to build a 16k library and to analyze a sample with 10^7^ reads (vs. 3 hours for krepp+DecoDiPhy with parallelization; Table S5). On more novel data (EMP), these methods that rely on exact k-mer matching fail to detect many relevant species, reducing their ability to characterize samples. Meanwhile, krepp and bowtie, which analyze reads individually, produce over-dispersed placements, decreasing accuracy and interpretability. DecoDiPhy consolidates these placements to a limited number of edges. On the IBD dataset, most DA features identified by DecoDiPhy are consistent with prior studies and with detections made by other tools. In addition, DecoDiPhy detects several internal and terminal features with limited or no known association to IBD, highlighting new targets for future investigation. When such features show high distance to samples (Fig. S11), our results highlight lack of sampling in the reference set.

Beyond metagenomics, PDD may find application elsewhere – identifying progenitor species of hybrids [6, 7, 62], distance-based admixture analysis on population trees [3–5, 108], and deconvolving cancer samples relative to existing ones [109]– topics left to future. Theoretical questions also remain, including characterizing unidentifiable cases, studying tolerance to error in $\overset{ˆ}{d}$ (analogous to near-additivity results [110]), and understanding optimality. We used a simple criterion to choose k, leaving more sophisticated stopping criteria for future work. Also, dynamic programming or ILP approaches may improve scalability for the exact algorithm, heuristic, or both. Finally, future work could consider alternatives distance calculation methods as input to PDDs, including Containment Jaccard [111] or distances computed by methods like sylph.

## Data Availability

The software is available at github.com/shayesteh99/DecoDiPhy. Data is available on Github github.com/shayesteh99/DecoDiPhy-Data.

## References

[1] Handelsman Jo. Metagenomics: application of genomics to uncultured microorganisms. Microbiology and molecular biology reviews, 68(4):669–85, December 2004. ISSN 1092-2172. doi: 10.1128/MMBR.68.4.669-685.2004. URL http://mmbr.asm.org/cgi/content/abstract/68/4/669. 15590779 PMC539003

[2] El-Kebir Mohammed, Oesper Layla, Acheson-Field Hannah, and Raphael Benjamin J.. Reconstruction of clonal trees and tumor composition from multi-sample sequencing data. Bioinformatics, 31(12): i62–i70, June 2015. ISSN 1367-4803, 1460-2059. doi: 10.1093/bioinformatics/btv261. 26072510 PMC4542783

[3] Kopelman Naama M., Stone Lewi, Gascuel Olivier, and Rosenberg Noah A.. The Behavior of Admixed Populations in Neighbor-Joining Inference of Population Trees. In Biocomputing 2013, pages 273–284, Kohala Coast, Hawaii, USA, November 2012. WORLD SCIENTIFIC. ISBN 978-981-4596-36-7 978-981-4447-97-3. doi: 10.1142/9789814447973_0027. URL http://www.worldscientific.com/doi/abs/10.1142/9789814447973_0027. PMC3597466 23424132

[4] Cheng Jade Yu, Mailund Thomas, and Nielsen Rasmus. Fast admixture analysis and population tree estimation for SNP and NGS data. Bioinformatics, 33(14):2148–2155, July 2017. ISSN 1367-4803, 1367-4811. doi: 10.1093/bioinformatics/btx098. URL https://academic.oup.com/bioinformatics/article/33/14/2148/3002763. 28334108 PMC6543773

[5] Pickrell Joseph and Pritchard Jonathan. Inference of population splits and mixtures from genome-wide allele frequency data. Nature Precedings, March 2012. ISSN 1756-0357. doi: 10.1038/npre.2012.6956.1. URL https://www.nature.com/articles/npre.2012.6956.1. PMC3499260 23166502

[6] Huson D. H.. SplitsTree: analyzing and visualizing evolutionary data. Bioinformatics, 14(1):68–73, 1998. ISSN 1367-4803. doi: 10.1093/bioinformatics/14.1.68. URL https://academic.oup.com/bioinformatics/article-lookup/doi/10.1093/bioinformatics/14.1.68. ISBN: 1367-4803 (Print). 9520503

[7] Langdon Quinn K, Peris David, Kyle Brian, and Hittinger Chris Todd. sppIDer: A Species Identification Tool to Investigate Hybrid Genomes with High-Throughput Sequencing. Molecular Biology and Evolution, September 2018. ISSN 0737-4038. doi: 10.1093/molbev/msy166. URL https://academic.oup.com/mbe/advance-article/doi/10.1093/molbev/msy166/5089242. PMC6231485 30184140

[8] Bastiaan von Meijenfeldt F. A., Arkhipova Ksenia, Cambuy Diego D., Coutinho Felipe H., and Dutilh Bas E.. Robust taxonomic classification of uncharted microbial sequences and bins with CAT and BAT. Genome Biology, 20(1):217, December 2019. ISSN 1474-760X. doi: 10.1186/s13059-019-1817-x. URL https://genomebiology.biomedcentral.com/articles/10.1186/s13059-019-1817-x. 31640809 PMC6805573

[9] Pachiadaki Maria G, Brown Julia M, Brown Joseph, Bezuidt Oliver, Berube Paul M, Biller Steven J, Poulton Nicole J, Burkart Michael D, La Clair James J, Chisholm Sallie W, and Stepanauskas Ramunas. Charting the Complexity of the Marine Microbiome through Single-Cell Genomics. Cell, 179(7):1623–1635.e11, 2019. ISSN 0092-8674. doi: 10.1016/j.cell.2019.11.017. URL http://www.sciencedirect.com/science/article/pii/S0092867419312735. 31835036 PMC6919566

[10] Rachtman Eleonora, Balaban Metin, Bafna Vineet, and Mirarab Siavash. The impact of contaminants on the accuracy of genome skimming and the effectiveness of exclusion read filters. Molecular Ecology Resources, 20(3):1755–0998.13135, May 2020. ISSN 1755-098X. doi: 10.1111/1755-0998.13135. URL https://onlinelibrary.wiley.com/doi/abs/10.1111/1755-0998.13135. 31943790

[11] Pasolli Edoardo, Asnicar Francesco, Manara Serena, Zolfo Moreno, Karcher Nicolai, Armanini Federica, Beghini Francesco, Manghi Paolo, Tett Adrian, Ghensi Paolo, Collado Maria Carmen, Rice Benjamin L., DuLong Casey, Morgan Xochitl C., Golden Christopher D., Quince Christopher, Huttenhower Curtis, and Segata Nicola. Extensive Unexplored Human Microbiome Diversity Revealed by Over 150,000 Genomes from Metagenomes Spanning Age, Geography, and Lifestyle. Cell, 176(3):649–662.e20, January 2019. ISSN 00928674. doi: 10.1016/j.cell.2019.01.001. URL https://linkinghub.elsevier.com/retrieve/pii/S0092867419300017. 30661755 PMC6349461

[12] Garmire Lana X., Li Yijun, Huang Qianhui, Xu Chuan, Teichmann Sarah A., Kaminski Naftali, Pellegrini Matteo, Nguyen Quan, and Teschendorff Andrew E.. Challenges and perspectives in computational deconvolution of genomics data. Nature Methods, 21(3):391–400, March 2024. ISSN 1548-7091, 1548-7105. doi: 10.1038/s41592-023-02166-6. URL https://www.nature.com/articles/s41592-023-02166-6. 38374264

[13] Schwartz Russell and Schäffer Alejandro A.. The evolution of tumour phylogenetics: principles and practice. Nature Reviews Genetics, 18(4):213–229, April 2017. ISSN 1471-0056, 1471-0064. doi: 10.1038/nrg.2016.170. PMC5886015 28190876

[14] Breitwieser Florian P, Lu Jennifer, and Salzberg Steven L. A review of methods and databases for metagenomic classification and assembly. Briefings in bioinformatics, 20(4):1125–1136, July 2019. ISSN 1477-4054 (Electronic). doi: 10.1093/bib/bbx120. URL http://www.sciencedirect.com/science/article/pii/S1055790316301488. 29028872 PMC6781581

[15] Knight Rob, Vrbanac Alison, Taylor Bryn C., Aksenov Alexander, Callewaert Chris, Debelius Justine, Gonzalez Antonio, Kosciolek Tomasz, McCall Laura-Isobel, McDonald Daniel, Melnik Alexey V., Morton James T., Navas Jose, Quinn Robert A., Sanders Jon G., Swafford Austin D., Thompson Luke R., Tripathi Anupriya, Xu Zhenjiang Z., Zaneveld Jesse R., Zhu Qiyun, Caporaso J. Gregory, and Dorrestein Pieter C.. Best practices for analysing microbiomes. Nature Reviews Microbiology, 16(7):410–422, July 2018. ISSN 1740-1526. doi: 10.1038/s41579-018-0029-9. URL http://www.nature.com/articles/s41579-018-0029-9. 29795328

[16] Callahan Benjamin J, McMurdie Paul J, Rosen Michael J, Han Andrew W, Johnson Amy Jo A, and Holmes Susan P. DADA2: High-resolution sample inference from Illumina amplicon data. Nature Methods, 13(7):581–583, July 2016. ISSN 1548-7091, 1548-7105. doi: 10.1038/nmeth.3869. URL https://www.nature.com/articles/nmeth.3869. 27214047 PMC4927377

[17] Zhu Qiyun, Huang Shi, Gonzalez Antonio, McGrath Imran, McDonald Daniel, Haiminen Niina, Armstrong George, Vázquez-Baeza Yoshiki, Yu Julian, Kuczynski Justin, Sepich-Poore Gregory D., Swafford Austin D., Das Promi, Shaffer Justin P., Lejzerowicz Franck, Belda-Ferre Pedro, Havulinna Aki S., Méric Guillaume, Niiranen Teemu, Lahti Leo, Salomaa Veikko, Kim Ho-Cheol, Jain Mohit, Inouye Michael, Gilbert Jack A., and Knight Rob. Phylogeny-Aware Analysis of Metagenome Community Ecology Based on Matched Reference Genomes while Bypassing Taxonomy. mSystems, 7(2):e0016722, April 2022. ISSN 2379-5077. doi: 10.1128/msystems.00167-22. 35369727 PMC9040630

[18] Schloss Patrick D. and Handelsman Jo. Introducing DOTUR, a Computer Program for Defining Operational Taxonomic Units and Estimating Species Richness. Applied and Environmental Microbiology, 71(3):1501–1506, March 2005. ISSN 0099-2240. doi: 10.1128/AEM.71.3.1501-1506.2005. URL https://aem.asm.org/content/71/3/1501. 15746353 PMC1065144

[19] O’Dwyer James P., Kembel Steven W., and Green Jessica L.. Phylogenetic Diversity Theory Sheds Light on the Structure of Microbial Communities. PLoS Computational Biology, 8(12):e1002832, December 2012. ISSN 1553-7358. doi: 10.1371/journal.pcbi.1002832. URL https://dx.plos.org/10.1371/journal.pcbi.1002832. ISBN: 1553-7358 (Electronic)\n1553-734X (Linking). 23284280 PMC3527210

[20] Washburne Alex D., Morton James T., Sanders Jon, McDonald Daniel, Zhu Qiyun, Oliverio Angela M., and Knight Rob. Methods for phylogenetic analysis of microbiome data. Nature Microbiology, 3(6): 652–661, May 2018. ISSN 2058-5276. doi: 10.1038/s41564-018-0156-0. URL https://www.nature.com/articles/s41564-018-0156-0. 29795540

[21] Matsen Frederick A. Phylogenetics and the Human Microbiome. Systematic Biology, 64(1):e26–e41, January 2015. ISSN 1076-836X. doi: 10.1093/sysbio/syu053. URL http://arxiv.org/abs/1407.1794. arXiv: 1407.1794. 25102857 PMC4265140

[22] Krause Lutz, Diaz Naryttza N, Goesmann Alexander, Kelley Scott, Nattkemper Tim W, Rohwer Forest, Edwards Robert A, and Stoye Jens. Phylogenetic classification of short environmental DNA fragments. Nucleic acids research, 36(7):2230–9, April 2008. ISSN 1362-4962. doi: 10.1093/nar/gkn038. URL http://nar.oxfordjournals.org/cgi/content/abstract/36/7/2230. 18285365 PMC2367736

[23] Asnicar Francesco, Thomas Andrew Maltez, Beghini Francesco, Mengoni Claudia, Manara Serena, Manghi Paolo, Zhu Qiyun, Bolzan Mattia, Cumbo Fabio, May Uyen, Sanders Jon G, Zolfo Moreno, Kopylova Evguenia, Pasolli Edoardo, Knight Rob, Mirarab Siavash, Huttenhower Curtis, and Segata Nicola. Precise phylogenetic analysis of microbial isolates and genomes from metagenomes using PhyloPhlAn 3.0. Nature Communications, 11(1):2500, December 2020. ISSN 2041-1723. doi: 10.1038/s41467-020-16366-7. URL http://dx.doi.org/10.1038/s41467-020-16366-7. Publisher: Springer US ISBN: 0348556977. PMC7237447 32427907

[24] Matsen Frederick A., Kodner Robin B., and Armbrust E. Virginia. pplacer: linear time maximum-likelihood and Bayesian phylogenetic placement of sequences onto a fixed reference tree. BMC Bioinformatics, 11(1):1–16, December 2010. ISSN 1471-2105. doi: 10.1186/1471-2105-11-538. URL https://bmcbioinformatics.biomedcentral.com/articles/10.1186/1471-2105-11-538. Number: 1 Publisher: BioMed Central. 21034504 PMC3098090

[25] Barbera Pierre, Kozlov Alexey M, Czech Lucas, Morel Benoit, Darriba Diego, Flouri Tomáš, and Stamatakis Alexandros. EPA-ng: Massively Parallel Evolutionary Placement of Genetic Sequences. Systematic Biology, 68(2):365–369, March 2019. ISSN 1063-5157. doi: 10.1093/sysbio/syy054. URL https://doi.org/10.1093/sysbio/syy054. 30165689 PMC6368480

[26] Wedell Eleanor, Shen Chengze, and Warnow Tandy. BSCAMPP: Batch-Scaled Phylogenetic Placement on Large Trees. IEEE Transactions on Computational Biology and Bioinformatics, 22(4):1593–1605, July 2025. ISSN 2998-4165. doi: 10.1109/TCBBIO.2025.3562281. URL https://ieeexplore.ieee.org/document/10969524/. 40811324

[27] Blanke Matthias and Morgenstern Burkhard. App-SpaM: phylogenetic placement of short reads without sequence alignment. Bioinformatics Advances, 1(1):vbab027, January 2021. ISSN 2635-0041. doi: 10.1093/bioadv/vbab027. URL https://doi.org/10.1093/bioadv/vbab027. 36700102 PMC9710606

[28] Mirarab Siavash, Nguyen Nam, and Warnow Tandy. SEPP: SATé-Enabled Phylogenetic Placement. In Pacific Symposium On Biocomputing, pages 247–58. WORLD SCIENTIFIC, December 2012. ISBN 978-981-4596-37-4. doi: 10.1142/9789814366496_0024. URL http://www.ncbi.nlm.nih.gov/pubmed/22174280. 22174280

[29] Sapci Ali Osman Berk and Mirarab Siavash. A k-mer-based maximum likelihood method for estimating distances of reads to genomes enables genome-wide phylogenetic placement. bioRxiv (RECOMB 2025), page 2025.01.20.633730, January 2025. doi: 10.1101/2025.01.20.633730. URL http://biorxiv.org/content/early/2025/01/24/2025.01.20.633730.abstract.

[30] Lozupone Catherine and Knight Rob. Unifrac: a new phylogenetic method for comparing microbial communities. Applied and Environmental Microbiology, 71(12):8228–8235, 2005. 16332807 10.1128/AEM.71.12.8228-8235.2005 PMC1317376

[31] Matsen Frederick A. and Evans Steven N.. Edge Principal Components and Squash Clustering: Using the Special Structure of Phylogenetic Placement Data for Sample Comparison. PLoS ONE, 8(3): e56859, March 2013. ISSN 1932-6203. doi: 10.1371/journal.pone.0056859. URL https://dx.plos.org/10.1371/journal.pone.0056859. arXiv: 1107.5095. 23505415 PMC3594297

[32] Ning Jie and Beiko Robert G.. Phylogenetic approaches to microbial community classification. Microbiome, 3(1):47, December 2015. ISSN 2049-2618. doi: 10.1186/s40168-015-0114-5. URL http://www.microbiomejournal.com/content/3/1/47. 26437943 PMC4593236

[33] Sayyari Erfan, Kawas Ban, and Mirarab Siavash. TADA: phylogenetic augmentation of microbiome samples enhances phenotype classification. Bioinformatics, 35(14):i31–i40, July 2019. ISSN 1367-4803. doi: 10.1093/bioinformatics/btz394. URL https://academic.oup.com/bioinformatics/article/35/14/i31/5529256. 31510701 PMC6612822

[34] Knights Dan, Kuczynski Justin, Koren Omry, Ley Ruth E, Field Dawn, Knight Rob, DeSantis Todd Z, and Kelley Scott T. Supervised classification of microbiota mitigates mislabeling errors. The ISME Journal, 5(4):570–573, April 2011. ISSN 1751-7362, 1751-7370. doi: 10.1038/ismej.2010.148. URL https://academic.oup.com/ismej/article/5/4/570/7587829. 20927137 PMC3105748

[35] Fioravanti Diego, Giarratano Ylenia, Maggio Valerio, Agostinelli Claudio, Chierici Marco, Jurman Giuseppe, and Furlanello Cesare. Phylogenetic convolutional neural networks in metagenomics. BMC Bioinformatics, 19(S2):49, March 2018. ISSN 1471-2105. doi: 10.1186/s12859-018-2033-5. URL https://bmcbioinformatics.biomedcentral.com/articles/10.1186/s12859-018-2033-5. 29536822 PMC5850953

[36] Wang Ye, Bhattacharya Tathagata, Jiang Yuchao, Qin Xiao, Wang Yue, Liu Yunlong, Saykin Andrew J, and Chen Li. A novel deep learning method for predictive modeling of microbiome data. Briefings in Bioinformatics, 22(3):bbaa073, May 2021. ISSN 1467-5463, 1477-4054. doi: 10.1093/bib/bbaa073. URL https://academic.oup.com/bib/article/doi/10.1093/bib/bbaa073/5835556. 32406914 PMC13017428

[37] Zhai Jing, Choi Youngwon, Yang Xingyi, Chen Yin, Knox Kenneth, Twigg Homer L., Won Joong-Ho, Zhou Hua, and Zhou Jin J.. DeepBiome: A Phylogenetic Tree Informed Deep Neural Network for Microbiome Data Analysis. Statistics in Biosciences, 17(1):191–215, April 2025. ISSN 1867-1764, 1867-1772. doi: 10.1007/s12561-024-09434-9. URL https://link.springer.com/10.1007/s12561-024-09434-9. 40894332 PMC12395559

[38] Morton James T., Sanders Jon, Quinn Robert A., McDonald Daniel, Gonzalez Antonio, Vázquez-Baeza Yoshiki, Navas-Molina Jose A., Song Se Jin, Metcalf Jessica L., Hyde Embriette R., Lladser Manuel, Dorrestein Pieter C., and Knight Rob. Balance Trees Reveal Microbial Niche Differentiation. mSystems, 2(1):e00162–16, February 2017. ISSN 2379-5077. doi: 10.1128/mSystems.00162-16. URL https://journals.asm.org/doi/10.1128/mSystems.00162-16. 28144630 PMC5264246

[39] Lin Huang and Peddada Shyamal Das. Analysis of microbial compositions: a review of normalization and differential abundance analysis. npj Biofilms and Microbiomes, 6(1):60, December 2020. ISSN 2055-5008. doi: 10.1038/s41522-020-00160-w. URL https://www.nature.com/articles/s41522-020-00160-w. 33268781 PMC7710733

[40] Liu Bo, Gibbons Theodore, Ghodsi Mohammad, and Pop Mihai. MetaPhyler: Taxonomic profiling for metagenomic sequences. In Bioinformatics and Biomedicine (BIBM), 2010 IEEE International Conference on, pages 95–100. IEEE, 2011. ISBN 978-1-4244-8305-1.

[41] Wood Derrick E., Lu Jennifer, and Langmead Ben. Improved metagenomic analysis with Kraken 2. Genome Biology, 20(1):257, November 2019. ISSN 1474-760X. doi: 10.1186/s13059-019-1891-0. URL https://doi.org/10.1186/s13059-019-1891-0. 31779668 PMC6883579

[42] Song Li and Langmead Ben. Centrifuger: lossless compression of microbial genomes for efficient and accurate metagenomic sequence classification. Genome Biology, 25(1):1–21, December 2024. ISSN 1474-760X. doi: 10.1186/s13059-024-03244-4. URL https://genomebiology.biomedcentral.com/articles/10.1186/s13059-024-03244-4. Number: 1 Publisher: BioMed Central. 38664753 PMC11046777

[43] Şapcı Ali Osman Berk, Rachtman Eleonora, and Mirarab Siavash. CONSULT-II: Accurate taxonomic identification and profiling using locality-sensitive hashing. Bioinformatics, page btae150, March 2024. ISSN 1367-4811. doi: 10.1093/bioinformatics/btae150. URL https://doi.org/10.1093/bioinformatics/btae150. 38492564 PMC10985673

[44] Piro Vitor C, Dadi Temesgen H, Seiler Enrico, Reinert Knut, and Renard Bernhard Y. ganon: precise metagenomics classification against large and up-to-date sets of reference sequences. Bioinformatics, 36(Supplement 1):i12–i20, July 2020. ISSN 1367-4803. doi: 10.1093/bioinformatics/btaa458. URL https://doi.org/10.1093/bioinformatics/btaa458. eprint: https://academic.oup.com/bioinformatics/article-pdf/36/Supplement_1/i12/57232196/bioinformatics_36_supplement1_i12.pdf. 32657362 PMC7355301

[45] Segata Nicola, Waldron Levi, Ballarini Annalisa, Narasimhan Vagheesh, Jousson Olivier, and Huttenhower Curtis. Metagenomic microbial community profiling using unique clade-specific marker genes. Nature Methods, 9(8):811–814, August 2012. ISSN 1548-7091. doi: 10.1038/nmeth.2066. URL http://www.nature.com/articles/nmeth.2066. ISBN: 1548-7091. 22688413 PMC3443552

[46] Shah Nidhi, Molloy Erin K, Pop Mihai, and Warnow Tandy. TIPP2: metagenomic taxonomic profiling using phylogenetic markers. Bioinformatics, 37(13):1839–1845, July 2021. ISSN 1367-4803. doi: 10.1093/bioinformatics/btab023. URL https://academic.oup.com/bioinformatics/article/37/13/1839/6104844. 33471121 PMC8317105

[47] Milanese Alessio, Mende Daniel R, Paoli Lucas, Salazar Guillem, Ruscheweyh Hans-Joachim, Cuenca Miguelangel, Hingamp Pascal, Alves Renato, Costea Paul I, Coelho Luis Pedro, Schmidt Thomas S B, Almeida Alexandre, Mitchell Alex L, Finn Robert D, Huerta-Cepas Jaime, Bork Peer, Zeller Georg, and Sunagawa Shinichi. Microbial abundance, activity and population genomic profiling with mOTUs2. Nature Communications, 10(1):1014, 2019. ISSN 2041-1723. doi: 10.1038/s41467-019-08844-4. URL https://doi.org/10.1038/s41467-019-08844-4. PMC6399450 30833550

[48] Nguyen Nam-phuong, Mirarab Siavash, Liu Bo, Pop Mihai, and Warnow Tandy. TIPP: taxonomic identification and phylogenetic profiling. Bioinformatics, 30(24):3548–3555, December 2014. ISSN 1460-2059. doi: 10.1093/bioinformatics/btu721. URL http://bioinformatics.oxfordjournals.org/cgi/doi/10.1093/bioinformatics/btu721. 25359891 PMC4253836

[49] Lu Jennifer, Breitwieser Florian P, Thielen Peter, and Salzberg Steven L. Bracken: estimating species abundance in metagenomics data. PeerJ Computer Science, 3:e104, January 2017. ISSN 2376-5992. doi: 10.7717/peerj-cs.104. URL https://doi.org/10.7717/peerj-cs.104. PMC12016282 40271438

[50] Koslicki David, Foucart Simon, and Rosen Gail. Quikr: A method for rapid reconstruction of bacterial communities via compressive sensing. Bioinformatics, 29(17):2096–2102, 2013. ISSN 13674803. doi: 10.1093/bioinformatics/btt336. 23786768

[51] Koslicki David and Falush Daniel. MetaPalette: a k-mer Painting Approach for Metagenomic Taxonomic Profiling and Quantification of Novel Strain Variation. mSystems, 1(3):1–18, June 2016. ISSN 2379-5077. doi: 10.1128/mSystems.00020-16. URL http://msystems.asm.org/lookup/doi/10.1128/mSystems.00020-16. PMC5069763 27822531

[52] Irber Luiz, Brooks Phillip T., Reiter Taylor, Pierce-Ward N. Tessa, Hera Mahmudur Rahman, Koslicki David, and Brown C. Titus. Lightweight compositional analysis of metagenomes with FracMinHash and minimum metagenome covers. preprint, Bioinformatics, January 2022. URL http://biorxiv.org/lookup/doi/10.1101/2022.01.11.475838.

[53] Ondov Brian D., Starrett Gabriel J., Sappington Anna, Kostic Aleksandra, Koren Sergey, Buck Christopher B., and Phillippy Adam M.. Mash Screen: high-throughput sequence containment estimation for genome discovery. Genome Biology, 20(1):1–13, December 2019. ISSN 1474-760X. doi: 10.1186/s13059-019-1841-x. URL https://genomebiology.biomedcentral.com/articles/10.1186/s13059-019-1841-x. Publisher: BioMed Central. 31690338 PMC6833257

[54] Shaw Jim and Yu Yun William. Rapid species-level metagenome profiling and containment estimation with sylph. Nature Biotechnology, 43(8):1348–1359, August 2025. ISSN 1546-1696. doi: 10.1038/s41587-024-02412-y. URL https://www.nature.com/articles/s41587-024-02412-y. Publisher: Nature Publishing Group. PMC12339375 39379646

[55] Foucart Simon and Koslicki David. Finer Metagenomic Reconstruction via Biodiversity Optimization, January 2020. URL http://biorxiv.org/lookup/doi/10.1101/2020.01.23.916924.

[56] Buneman Peter. A note on the metric properties of trees. Journal of Combinatorial Theory, Series B, 17(1):48–50, 1974. ISSN 00958956. doi: 10.1016/0095-8956(74)90047-1.

[57] Fitch Walter M. and Margoliash Emanuel. Construction of Phylogenetic Trees. Science, 155(3760): 279–284, January 1967. ISSN 0036-8075. doi: 10.1126/science.155.3760.279. URL https://www.science.org/doi/10.1126/science.155.3760.279. 5334057

[58] Saitou N and Nei Masatoshi. The neighbour-joining method: a new method for reconstructing phylogenetic trees. Molecular biology and evolution, 4(4):406–425, 1987. 3447015 10.1093/oxfordjournals.molbev.a040454

[59] Desper Richard and Gascuel Olivier. Fast and Accurate Phylogeny Reconstruction Algorithms Based on the Minimum-Evolution Principle. Journal of Computational Biology, 9(5):687–705, October 2002. ISSN 1066-5277. doi: 10.1089/106652702761034136. URL http://www.liebertonline.com/doi/abs/10.1089/106652702761034136. 12487758

[60] Balaban Metin, Sarmashghi Shahab, and Mirarab Siavash. APPLES: Scalable Distance-Based Phylogenetic Placement with or without Alignments. Systematic Biology, 69(3):566–578, May 2020. ISSN 1063-5157. doi: 10.1093/sysbio/syz063. URL https://academic.oup.com/sysbio/advance-article/doi/10.1093/sysbio/syz063/5572672. 31545363 PMC7164367

[61] Matsen Frederick A, Kodner Robin B, and Armbrust E Virginia. pplacer: linear time maximum-likelihood and Bayesian phylogenetic placement of sequences onto a fixed reference tree. BMC bioinformatics, 11(1):538, October 2010. ISSN 1471-2105. doi: 10.1186/1471-2105-11-538. URL http://www.pubmedcentral.nih.gov/articlerender.fcgi?artid=3098090&tool=pmcentrez&rendertype=abstract. 21034504 PMC3098090

[62] Balaban Metin and Mirarab Siavash. Phylogenetic double placement of mixed samples. Bioinformatics, 36(Supplement 1):i335–i343, July 2020. ISSN 1367-4803. doi: 10.1093/bioinformatics/btaa489. URL https://academic.oup.com/bioinformatics/article/36/Supplement_1/i335/5870522. 32657414 PMC7355250

[63] Warnow Tandy. Computational phylogenetics: An introduction to designing methods for phylogeny estimation. Cambridge University Press, Cambridge, United Kingdom, 2017. ISBN 1-316-88692-1. Address: Cambridge, United Kingdom.

[64] Diamond Steven and Boyd Stephen. CVXPY: A Python-embedded modeling language for convex optimization. Journal of Machine Learning Research, 2016. URL https://stanford.edu/~boyd/papers/pdf/cvxpy_paper.pdf. PMC4927437 27375369

[65] Stellato B., Banjac G., Goulart P., Bemporad A., and Boyd S.. OSQP: an operator splitting solver for quadratic programs. Mathematical Programming Computation, 12(4):637–672, 2020. doi: 10.1007/s12532-020-00179-2. URL https://doi.org/10.1007/s12532-020-00179-2.

[66] Jukes T H and Cantor C R. Evolution of protein molecules. Mammalian protein metabolism, 3:21–132, 1969. doi: 10.1234/12345678.

[67] Huang Weichun, Li Leping, Myers Jason R, and Marth Gabor T. ART: A next-generation sequencing read simulator. Bioinformatics, 28(4):593–594, 2012. ISSN 13674803. doi: 10.1093/bioinformatics/btr708. 22199392 PMC3278762

[68] Şapcı Ali Osman Berk and Mirarab Siavash. A k-mer-based maximum likelihood method for estimating distances of reads to genomes enables genome-wide phylogenetic placement. bioRxiv; doi: 10.1101/2025.01.20.633730. PMC13032499 41723514

[69] Balaban Metin, Jiang Yueyu, Zhu Qiyun, McDonald Daniel, Knight Rob, and Mirarab Siavash. Generation of accurate, expandable phylogenomic trees with uDance. Nature Biotechnology, Online, July 2023. ISSN 1546-1696. doi: 10.1038/s41587-023-01868-8. URL https://doi.org/10.1038/s41587-023-01868-8. PMC10818028 37500914

[70] Langmead Ben and Salzberg Steven L.. Fast gapped-read alignment with Bowtie 2. Nature Methods, 9 (4):357–359, April 2012. ISSN 1548-7105. doi: 10.1038/nmeth.1923. URL https://www.nature.com/articles/nmeth.1923. Publisher: Nature Publishing Group. 22388286 PMC3322381

[71] Roger Bray J. and Curtis J. T.. An ordination of the upland forest communities of southern wisconsin. Ecological Monographs, 27(4):325–349, 1957.

[72] Lozupone Catherine A., Hamady Micah, Kelley Scott T., and Knight Rob. Quantitative and qualitative diversity measures lead to different insights into factors that structure microbial communities. Applied and Environmental Microbiology, 73(5):1576–1585, 2007. 17220268 10.1128/AEM.01996-06 PMC1828774

[73] Franzosa Eric A., Sirota-Madi Alexandra, Avila-Pacheco Julian, Fornelos Nadine, Haiser Henry J., Reinker Stefan, Vatanen Tommi, Hall A. Brantley, Mallick Himel, McIver Lauren J., Sauk Jenny S., Wilson Robin G., Stevens Betsy W., Scott Justin M., Pierce Kerry, Deik Amy A., Bullock Kevin, Imhann Floris, Porter Jeffrey A., Zhernakova Alexandra, Fu Jingyuan, Weersma Rinse K., Wijmenga Cisca, Clish Clary B., Vlamakis Hera, Huttenhower Curtis, and Xavier Ramnik J.. Gut microbiome structure and metabolic activity in inflammatory bowel disease. Nature Microbiology, 4(2):293–305, December 2018. ISSN 2058-5276. doi: 10.1038/s41564-018-0306-4. URL https://www.nature.com/articles/s41564-018-0306-4. PMC6342642 30531976

[74] Shaffer Justin P., Nothias Louis-Félix, Thompson Luke R., Sanders Jon G., Salido Rodolfo A., Couvillion Sneha P., Brejnrod Asker D., Lejzerowicz Franck, Haiminen Niina, Huang Shi, Lutz Holly L., Zhu Qiyun, Martino Cameron, Morton James T., Karthikeyan Smruthi, Nothias-Esposito Mélissa, Dührkop Kai, Böcker Sebastian, Kim Hyun Woo, Aksenov Alexander A., Bittremieux Wout, Minich Jeremiah J., Marotz Clarisse, Bryant MacKenzie M., Sanders Karenina, Schwartz Tara, Humphrey Greg, Vásquez-Baeza Yoshiki, Tripathi Anupriya, Parida Laxmi, Carrieri Anna Paola, Beck Kristen L., Das Promi, González Antonio, McDonald Daniel, Ladau Joshua, Karst Søren M., Albertsen Mads, Ackermann Gail, DeReus Jeff, Thomas Torsten, Petras Daniel, Shade Ashley, Stegen James, Song Se Jin, Metz Thomas O., Swafford Austin D., Dorrestein Pieter C., Jansson Janet K., Gilbert Jack A., Knight Rob, the Earth Microbiome Project 500 (EMP500) Consortium, Angenant Lars T., Berry Alison M., Bittleston Leonora S., Bowen Jennifer L., Chavarría Max, Cowan Don A., Distel Dan, Girguis Peter R., Huerta-Cepas Jaime, Jensen Paul R., Jiang Lingjing, King Gary M., Lavrinienko Anton, MacRae-Crerar Aurora, Makhalanyane Thulani P., Mappes Tapio, Marzinelli Ezequiel M., Mayer Gregory, McMahon Katherine D., Metcalf Jessica L., Miyake Sou, Mousseau Timothy A., Murillo-Cruz Catalina, Myrold David, Palenik Brian, Pinto-Tomás Adrián A., Porazinska Dorota L., Ramond Jean-Baptiste, Rowher Forest, RoyChowdhury Taniya, Sandin Stuart A., Schmidt Steven K., Seedorf Henning, Shade Ashley, Shipway J. Reuben, Smith Jennifer E., Stegen James, Stewart Frank J., Tait Karen, Thomas Torsten, Tucker Yael, U’Ren Jana M., Watts Phillip C., Webster Nicole S., Zaneveld Jesse R., and Zhang Shan. Standardized multi-omics of Earth’s microbiomes reveals microbial and metabolite diversity. Nature Microbiology, 7(12):2128–2150, November 2022. ISSN 2058-5276. doi: 10.1038/s41564-022-01266-x. URL https://www.nature.com/articles/s41564-022-01266-x. PMC9712116 36443458

[75] Bolyen Evan, Jai Ram Rideout Matthew R. Dillon, Bokulich Nicholas A., Abnet Christian C., Al-Ghalith Gabriel A., Alexander Harriet, Alm Eric J., Arumugam Manimozhiyan, Asnicar Francesco, Bai Yang, Bisanz Jordan E., Bittinger Kyle, Brejnrod Asker, Brislawn Colin J., Brown C. Titus, Callahan Benjamin J., Caraballo-Rodríguez Andrés Mauricio, Chase John, Cope Emily K., Silva Ricardo Da, Diener Christian, Dorrestein Pieter C., Douglas Gavin M., Durall Daniel M., Duvallet Claire, Edwardson Christian F., Ernst Madeleine, Estaki Mehrbod, Fouquier Jennifer, Gauglitz Julia M., Gibbons Sean M., Gibson Deanna L., Gonzalez Antonio, Gorlick Kestrel, Guo Jiarong, Hillmann Benjamin, Holmes Susan, Holste Hannes, Huttenhower Curtis, Huttley Gavin A., Janssen Stefan, Jarmusch Alan K., Jiang Lingjing, Kaehler Benjamin D., Kang Kyo Bin, Keefe Christopher R., Keim Paul, Kelley Scott T., Knights Dan, Koester Irina, Kosciolek Tomasz, Kreps Jorden, Langille Morgan G. I., Lee Joslynn, Ley Ruth, Liu Yong-Xin, Loftfield Erikka, Lozupone Catherine, Maher Massoud, Marotz Clarisse, Martin Bryan D., McDonald Daniel, McIver Lauren J., Melnik Alexey V., Metcalf Jessica L., Morgan Sydney C., Morton Jamie T., Naimey Ahmad Turan, Navas-Molina Jose A., Nothias Louis Felix, Orchanian Stephanie B., Pearson Talima, Peoples Samuel L., Petras Daniel, Preuss Mary Lai, Pruesse Elmar, Rasmussen Lasse Buur, Rivers Adam, Robeson Michael S., Rosenthal Patrick, Segata Nicola, Shaffer Michael, Shiffer Arron, Sinha Rashmi, Song Se Jin, Spear John R., Swafford Austin D., Thompson Luke R., Torres Pedro J., Trinh Pauline, Tripathi Anupriya, Turnbaugh Peter J., Ul-Hasan Sabah, van der Hooft Justin J. J., Vargas Fernando, Vázquez-Baeza Yoshiki, Vogtmann Emily, von Hippel Max, Walters William, Wan Yunhu, Wang Mingxun, Warren Jonathan, Weber Kyle C., Williamson Charles H. D., Willis Amy D., Xu Zhenjiang Zech, Zaneveld Jesse R., Zhang Yilong, Zhu Qiyun, Knight Rob, and Caporaso J. Gregory. Reproducible, interactive, scalable and extensible microbiome data science using qiime 2. Nature Biotechnology, 37(8):852–857, 2019. ISSN 1546-1696. doi: 10.1038/s41587-019-0209-9. URL https://doi.org/10.1038/s41587-019-0209-9. PMC7015180 31341288

[76] Vacca Mirco, Celano Giuseppe, Francesco Maria Calabrese Piero Portincasa, Gobbetti Marco, and De Angelis Maria. The controversial role of human gut lachnospiraceae. Microorganisms, 8(4), Apr 2020. 10.3390/microorganisms8040573 PMC7232163 32326636

[77] Singh Vineet, Lee GyuDae, Son HyunWoo, Koh Hong, Kim Eun Soo, Unno Tatsuya, and Shin Jae-Ho. Butyrate producers, “the sentinel of gut”: Their intestinal significance with and beyond butyrate, and prospective use as microbial therapeutics. Front Microbiol, 13:1103836, 2022. 36713166 10.3389/fmicb.2022.1103836 PMC9877435

[78] Vestergaard Marie Vibeke, Allin Kristine H., Eriksen Carsten, Zakerska-Banaszak Oliwia, Arasaradnam Ramesh P., Alam Mohammad T., Kristiansen Karsten, Brix Susanne, and Jess Tine. Gut microbiota signatures in inflammatory bowel disease. United European Gastroenterology Journal, 12(1):22–33, 2024. 38041519 10.1002/ueg2.12485 PMC10859715

[79] Sharma Bharti, Agriantonis George, Twelker Kate, Ebelle Danielle, Kiernan Samantha, Siddiqui Maham, Soni Aditi, Cheerasarn Sittha, Simon Whenzdjyny, Jiang Winston, Cardona Angie, Chapelet Jessica, Agathis Alexandra Z., Gamboa Alejandro, Dave Jasmine, Mestre Juan, Bhatia Navin D., Shaefee Zahra, and Whittington Jennifer. Gut microbiota serves as a crucial independent biomarker in inflammatory bowel disease (ibd). International Journal of Molecular Sciences, 26(6), 2025. 40141145 10.3390/ijms26062503 PMC11942158

[80] Sokol Harry, Pigneur Bénédicte, Watterlot Laurie, Lakhdari Omar, Bermúdez-Humarán Luis G., Gratadoux JeanJacques, Blugeon Sébastien, Bridonneau Chantal, Furet Jean-Pierre, Corthier Gérard, Grangette Corinne, Vasquez Nadia, Pochart Philippe, Trugnan Germain, Thomas Ginette, Blottière Hervé M., Doré Joël, Marteau Philippe, Seksik Philippe, and Langella Philippe. ¡i¿faecalibacterium prausnitzii¡/i¿ is an anti-inflammatory commensal bacterium identified by gut microbiota analysis of crohn disease patients. Proceedings of the National Academy of Sciences, 105(43):16731–16736, 2008. 10.1073/pnas.0804812105 PMC2575488 18936492

[81] Dahal Ram Hari, Kim Shukho, Kim Yu Kyung, Kim Eun Soo, and Kim Jungmin. Insight into gut dysbiosis of patients with inflammatory bowel disease and ischemic colitis. Front Microbiol, 14:1174832, 2023. 37250025 10.3389/fmicb.2023.1174832 PMC10211348

[82] Li Junhui and O’Toole Paul W. Disease-associated microbiome signature species in the gut. PNAS Nexus, 3(9):pgae352, 08 2024. 10.1093/pnasnexus/pgae352 PMC11370893 39228810

[83] Sankarasubramanian Jagadesan, Ahmad Rizwan, Avuthu Nagavardhini, Singh Amar B, and Guda Chittibabu. Gut microbiota and metabolic specificity in ulcerative colitis and crohn’s disease. Front Med (Lausanne), 7:606298, 2020. 33330572 10.3389/fmed.2020.606298 PMC7729129

[84] Ma Xianzong, Lu Xiaojuan, Zhang Wenyu, Yang Lang, Wang Dezhi, Xu Junfeng, Jia Yan, Wang Xin, Xie Hui, Li Shu, Zhang Mingjie, He Yuqi, Jin Peng, and Sheng Jianqiu. Gut microbiota in the early stage of crohn’s disease has unique characteristics. Gut Pathogens, 14(1):46, 2022. 36517872 10.1186/s13099-022-00521-0 PMC9753350

[85] Geese Theresa, Bang Corinna, Franke Andre, Lieb Wolfgang, and Dempfle Astrid. The human gut microbiota in ibd, characterizing hubs, the core microbiota and terminal nodes: a network-based approach. BMC Microbiology, 25(1):371, 2025. 40571931 10.1186/s12866-025-04106-0 PMC12199529

[86] Rajilić-Stojanović Mirjana, Biagi Elena, Heilig Hans G. H. J., Kajander Kajsa, Kekkonen Riina A., Tims Sebastian, and de Vos Willem M.. Global and deep molecular analysis of microbiota signatures in fecal samples from patients with irritable bowel syndrome. Gastroenterology, 141(5):1792–1801, 2025/11/12 2011. 21820992 10.1053/j.gastro.2011.07.043

[87] Nishino Kyohei, Nishida Atsushi, Inoue Ryo, Kawada Yuki, Ohno Masashi, Sakai Shigeki, Inatomi Osamu, Bamba Shigeki, Sugimoto Mitsushige, Kawahara Masahiro, Naito Yuji, and Andoh Akira. Analysis of endoscopic brush samples identified mucosa-associated dysbiosis in inflammatory bowel disease. Journal of Gastroenterology, 53(1):95–106, 2018. 28852861 10.1007/s00535-017-1384-4

[88] Caruso Roberta, Lo Bernard C., and Núñez Gabriel. Host–microbiota interactions in inflammatory bowel disease. Nature Reviews Immunology, 20(7):411–426, 2020. 10.1038/s41577-019-0268-7 32005980

[89] Palmieri Orazio, Bossa Fabrizio, Castellana Stefano, Latiano Tiziana, Carparelli Sonia, Martino Giuseppina, Mangoni Manuel, Corritore Giuseppe, Nardella Marianna, Guerra Maria, Biscaglia Giuseppe, Perri Francesco, Mazza Tommaso, and Latiano Anna. Deciphering microbial composition in patients with inflammatory bowel disease: Implications for therapeutic response to biologic agents. Microorganisms, 12(7), 2024. 39065032 10.3390/microorganisms12071260 PMC11278628

[90] Pisani Anthea, Rausch Philipp, Bang Corinna, Ellul Sarah, Tabone Trevor, Cordina Claire Marantidis, Zahra Graziella, Franke Andre, and Ellul Pierre. Dysbiosis in the gut microbiota in patients with inflammatory bowel disease during remission. Microbiol Spectr, 10(3):e0061622, Jun 2022. 35532243 10.1128/spectrum.00616-22 PMC9241752

[91] Blankenberger Amanda, Lesmana Elvira, Yang Lu, Comba Isin Y., Edwinson Adam, BreenLyles Margaret, Keehn Ashley, Patel Robin, Chen Jun, Mars Ruben A., Kashyap Purna, Farrugia Gianrico, and Grover Madhusudan. Longitudinal changes in the gut microbiome of patients with irritable bowel syndrome after ¡em¿campylobacter¡/em¿ infection are associated with proteolytic activity. Gastro Hep Advances, 4(8), 2025/11/05 2025. 10.1016/j.gastha.2025.100683 PMC12166722 40520420

[92] Li Congxin, Peng Kaixin, Xiao Siqi, Long Yuanyuan, and Yu Qin. The role of lactobacillus in inflammatory bowel disease: from actualities to prospects. Cell Death Discov, 9(1):361, Sep 2023. 37773196 10.1038/s41420-023-01666-w PMC10541886

[93] Khusnutdinova D, Markelova M, Siniagina M, Boulygina E, Abdulkhakov S, and Grigoryeva T. P053 overabundance of lactobacillus species in gut microbiota of ibd patients. Journal of Crohn’s and Colitis, 15:S160–S161, 05 2021.

[94] Kushkevych Ivan, Dordević Dani, and Kollár Peter. Analysis of physiological parameters of desulfovibrio strains from individuals with colitis. Open Life Sci, 13:481–488, Jan 2018. 33817117 10.1515/biol-2018-0057 PMC7874683

[95] Metwaly Amira, Dunkel Andreas, Waldschmitt Nadine, Durai Raj Abilash Chakravarthy, Lagkouvardos Ilias, Corraliza Ana Maria, Mayorgas Aida, Martinez-Medina Margarita, Reiter Sinah, Schloter Michael, Hofmann Thomas, Allez Matthieu, Panes Julian, Salas Azucena, and Haller Dirk. Integrated microbiota and metabolite profiles link crohn’s disease to sulfur metabolism. Nat Commun, 11(1): 4322, Aug 2020. 32859898 10.1038/s41467-020-17956-1 PMC7456324

[96] Coutinho Cláudia Mara Lara Melo, Coutinho-Silva Robson, Zinkevich Vitally, Pearce Callum B., Ojcius David M., and Beech Iwona. Sulphate-reducing bacteria from ulcerative colitis patients induce apoptosis of gastrointestinal epithelial cells. Microbial Pathogenesis, 112:126–134, 2017. 28963010 10.1016/j.micpath.2017.09.054

[97] Jalandra R, Dhar R, Pethusamy K, Sharma M, and Karmakar S. Dysbiosis: Gut feeling [version 1; peer review: awaiting peer review]. F1000Research, 11(911), 2022.

[98] Jangid Aditi, Fukuda Shinji, Seki Masahide, Horiuchi Terumi, Suzuki Yutaka, Taylor Todd D, Ohno Hiroshi, and Prakash Tulika. Association of colitis with gut-microbiota dysbiosis in clathrin adapter ap-1b knockout mice. PLoS One, 15(3):e0228358, 2020. 32208434 10.1371/journal.pone.0228358 PMC7093000

[99] Gao Lei, Narsing Rao Manik Prabhu, Liu Yong-Hong, Wang Pan-Deng, Lian Zheng-Han, Abdugheni Rashidin, Jiang Hong-Chen, Jiao Jian-Yu, Shurigin Vyacheslav, Fang Bao-Zhu, and Li Wen-Jun. Salinity-induced changes in diversity, stability, and functional profiles of microbial communities in different saline lakes in arid areas. Microbial Ecology, 87(1):135, 2024. 39482450 10.1007/s00248-024-02442-8 PMC11527964

[100] Zhang H., Chang D., Zhu Z., Meng C., and Wang K.. Soil priming effects and involved microbial community along salt gradients. Biogeosciences, 21(1):1–11, 2024.

[101] Mucsi Márton, Borsodi Andrea K, Megyes Melinda, and Szili-Kovács Tibor. Response of the metabolic activity and taxonomic composition of bacterial communities to mosaically varying soil salinity and alkalinity. Sci Rep, 14(1):7460, Mar 2024. 38553497 10.1038/s41598-024-57430-2 PMC10980690

[102] Ma Bin and Gong Jun. A meta-analysis of the publicly available bacterial and archaeal sequence diversity in saline soils. World Journal of Microbiology and Biotechnology, 29(12):2325–2334, 2013. 23756871 10.1007/s11274-013-1399-9

[103] Wang Shuang, Sun Lei, Ling Ning, Zhu Chen, Chi Fengqin, Li Weiqun, Hao Xiaoyu, Zhang Wu, Bian Jingyang, Chen Lei, and Wei Dan. Exploring soil factors determining composition and structure of the bacterial communities in saline-alkali soils of songnen plain. Frontiers in Microbiology, Volume 10 – 2019, 2020. 10.3389/fmicb.2019.02902 PMC6972583 32010065

[104] Lozupone Catherine A and Knight Rob. Global patterns in bacterial diversity. Proc Natl Acad Sci U S A, 104(27):11436–11440, Jul 2007. 17592124 10.1073/pnas.0611525104 PMC2040916

[105] Herlemann Daniel PR, Labrenz Matthias, Jürgens Klaus, Bertilsson Stefan, Waniek Joanna J, and Andersson Anders F. Transitions in bacterial communities along the 2000 km salinity gradient of the baltic sea. The ISME Journal, 5(10):1571–1579, 2011. 21472016 10.1038/ismej.2011.41 PMC3176514

[106] Herlemann Daniel P R, Lundin Daniel, Andersson Anders F, Labrenz Matthias, and Jürgens Klaus. Phylogenetic signals of salinity and season in bacterial community composition across the salinity gradient of the baltic sea. Front Microbiol, 7:1883, 2016. 27933046 10.3389/fmicb.2016.01883 PMC5121245

[107] Janssen Stefan, McDonald Daniel, Gonzalez Antonio, Navas-Molina Jose A., Jiang Lingjing, Xu Zhenjiang Zech, Winker Kevin, Kado Deborah M., Orwoll Eric, Manary Mark, Mirarab Siavash, and Knight Rob. Phylogenetic Placement of Exact Amplicon Sequences Improves Associations with Clinical Information. mSystems, 3(3):e00021–18, April 2018. ISSN 2379-5077. doi: 10.1128/mSystems.00021-18. URL http://msystems.asm.org/lookup/doi/10.1128/mSystems.00021-18. 29719869 PMC5904434

[108] Kim Jaehee, Disanto Filippo, Kopelman Naama M., and Rosenberg Noah A.. Mathematical and Simulation-Based Analysis of the Behavior of Admixed Taxa in the Neighbor-Joining Algorithm. Bulletin of Mathematical Biology, 81(2):452–493, February 2019. ISSN 0092-8240, 1522-9602. doi: 10.1007/s11538-018-0444-0. URL http://link.springer.com/10.1007/s11538-018-0444-0. 29876842 PMC7723469

[109] El-Kebir Mohammed, Satas Gryte, and Raphael Benjamin J.. Inferring parsimonious migration histories for metastatic cancers. Nature Genetics, 50(5):718–726, May 2018. ISSN 1061-4036, 1546-1718. doi: 10.1038/s41588-018-0106-z. 29700472 PMC6103651

[110] Atteson K.. The Performance of Neighbor-Joining Methods of Phylogenetic Reconstruction. Algorithmica, 25(2–3):251–278, 1999. ISSN 0178-4617. doi: 10.1007/PL00008277.

[111] Koslicki David and Zabeti Hooman. Improving MinHash via the containment index with applications to metagenomic analysis. Applied Mathematics and Computation, 354:206–215, August 2019. ISSN 00963003. doi: 10.1016/j.amc.2019.02.018. URL https://linkinghub.elsevier.com/retrieve/pii/S009630031930116X.

[112] Jiang Yueyu, Balaban Metin, Zhu Qiyun, and Mirarab Siavash. DEPP: Deep Learning Enables Extending Species Trees using Single Genes. Systematic Biology, 72(1):17–34, April 2022. ISSN 1063-5157. doi: 10.1093/sysbio/syac031. URL https://academic.oup.com/sysbio/advance-article/doi/10.1093/sysbio/syac031/6575921. PMC10198656 35485976

[113] Ze Xiaolei, Duncan Sylvia H, Louis Petra, and Flint Harry J. Ruminococcus bromii is a keystone species for the degradation of resistant starch in the human colon. ISME J, 6(8):1535–1543, Aug 2012. 22343308 10.1038/ismej.2012.4 PMC3400402

[114] Kang Seungha, Denman Stuart E., Morrison Mark, Yu Zhongtang, Dore Joel, Leclerc Marion, and McSweeney Chris S.. Dysbiosis of fecal microbiota in crohn’s disease patients as revealed by a custom phylogenetic microarray. Inflammatory Bowel Diseases, 16(12):2034–2042, 05 2010. 20848492 10.1002/ibd.21319

[115] Iliev Iliyan D., Ananthakrishnan Ashwin N., and Guo Chun-Jun. Microbiota in inflammatory bowel disease: mechanisms of disease and therapeutic opportunities. Nature Reviews Microbiology, 23(8): 509–524, 2025. 40065181 10.1038/s41579-025-01163-0 PMC12289240

[116] Ning Lijun, Zhou Yi-Lu, Sun Han, Zhang Youwei, Shen Chaoqin, Wang Zhenhua, Xuan Baoqin, Zhao Ying, Ma Yanru, Yan Yuqing, Tong Tianying, Huang Xiaowen, Hu Muni, Zhu Xiaoqiang, Ding Jinmei, Zhang Yue, Cui Zhe, Fang Jing-Yuan, Chen Haoyan, and Hong Jie. Microbiome and metabolome features in inflammatory bowel disease via multi-omics integration analyses across cohorts. Nature Communications, 14(1):7135, 2023. 10.1038/s41467-023-42788-0 PMC10628233 37932270

[117] Mayorga Luis, Serrano-Gómez Gerard, Xie Zixuan, Borruel Natalia, and Manichanh Chaysavanh. Intercontinental gut microbiome variances in ibd. Int J Mol Sci, 23(18), Sep 2022. 10.3390/ijms231810868 PMC9506019 36142786

[118] Song Han, Yoo Young, Hwang Junghyun, Na Yun-Cheol, and Kim Heenam Stanley. ¡em¿faecalibacterium prausnitzii¡/em¿ subspecies–level dysbiosis in the human gut microbiome underlying atopic dermatitis. Journal of Allergy and Clinical Immunology, 137(3):852–860, 2025/10/28 2016. 26431583 10.1016/j.jaci.2015.08.021

[119] Mclellan Paul, Auger Sandrine, Goudiaby Marie Therese, Brot Loic, Benech Nicolas, Grill Jean Pierre, Bourrier Anne, Mariat Denis, Mayeur Camille, Thomas Muriel, Robert Véronique, Kirchgesner Julien, Beaugerie Laurent, Sokol Harry, Langella Philippe, Seksik Philippe, and Chatel Jean Marc. Faecalibacterium diversity in the gut microbiome of crohn’s disease patients. United European Gastroenterol J, 13(8):1480–1490, Oct 2025. 10.1002/ueg2.70023 PMC12529001 40252217

[120] Zhao Shidong, Zheng Xianjing, Yang Changjiang, Shen Weisong, and Shen Zhanlong. Gut microbiota causally affects ulcerative colitis by potential mediation of plasma metabolites: A mendelian randomization study. Medicine (Baltimore), 104(26):e42791, Jun 2025. 40587710 10.1097/MD.0000000000042791 PMC12212764

[121] Takahashi Kenichiro, Nishida Atsushi, Fujimoto Takehide, Fujii Makoto, Shioya Makoto, Imaeda Hirotsugu, Inatomi Osamu, Bamba Shigeki, Andoh Akira, and Sugimoto Mitsushige. Reduced abundance of butyrate-producing bacteria species in the fecal microbial community in crohn’s disease. Digestion, 93(1):59–65, 01 2016. 26789999 10.1159/000441768

[122] Oñate Florian Plaza, Chamignon Célia, Burz Sebastian D, Lapaque Nicolas, Monnoye Magali, Philippe Catherine, Bredel Maxime, Chêne Laurent, Farin William, Paillarse Jean-Michel, Boursier Jérome, Ratziu Vlad, Mousset Pierre-Yves, Doré Joël, Gérard Philippe, and Blottière HervéM. Adlercreutzia equolifaciens is an anti-inflammatory commensal bacterium with decreased abundance in gut microbiota of patients with metabolic liver disease. Int J Mol Sci, 24(15), Jul 2023. 10.3390/ijms241512232 PMC10418321 37569608

[123] Galipeau Heather J., Caminero Alberto, Turpin Williams, Bermudez-Brito Miriam, Santiago Alba, Libertucci Josie, Constante Marco, Garay Juan Antonio Raygoza, Rueda Gaston, Armstrong Sarah, Clarizio Alex, Smith Michelle I., Surette Michael G., Bercik Premysl, Beck Paul, Bernstein Charles, Croitoru Kenneth, Dieleman Leo, Feagan Brian, Griffiths Anne, Guttman David, Jacobson Kevan, Kaplan Gilaad, Krause Denis O., Madsen Karen, Marshall John, Moayyedi Paul, Ropeleski Mark, Seidman Ernest, Silverberg Mark, Snapper Scott, Stadnyk Andy, Steinhart Hillary, Surette Michael, Turner Dan, Walters Thomas, Vallance Bruce, Aumais Guy, Bitton Alain, Cino Maria, Critch Jeff, Denson Lee, Deslandres Colette, El-Matary Wael, Herfarth Hans, Higgins Peter, Huynh Hien, Hyams Jeff, Mack David, McGrath Jerry, Otley Anthony, Panancionne Remo, and Verdu Elena F.. Novel fecal biomarkers that precede clinical diagnosis of ulcerative colitis. Gastroenterology, 160(5):1532–1545, 2025/10/29 2021. 33310084 10.1053/j.gastro.2020.12.004

[124] Zhang Zeng, Yang Zhihan, Lin Shijia, Jiang Shuaiming, Zhou Xiaolu, Li Jiahe, Lu Wei, and Zhang Jiachao. Probiotic-induced enrichment of adlercreutzia equolifaciens increases gut microbiome wellness index and maps to lower host blood glucose levels. Gut Microbes, 17(1):2520407, Dec 2025. 40515809 10.1080/19490976.2025.2520407 PMC12169038

[125] Rohmann Nathalie, Geese Theresa, Nestel Samantha, Schlicht Kristina, Geisler Corinna, Türk Kathrin, Brix Fynn, Jensen-Kroll Julia, Demetrowitsch Tobias, Bang Corinna, Franke Andre, Lieb Wolfgang, Schulte Dominik M., Schwarz Karin, Ruß Anne-Kathrin, Sharma Arunabh, Schreiber Stefan, Dempfle Astrid, and Laudes Matthias. Metabolic and lifestyle factors accelerate disease onset and alter gut microbiome in inflammatory non-communicable diseases. BMC Medicine, 22(1):493, 2024. 39449123 10.1186/s12916-024-03709-0 PMC11515311

[126] Park Sunmin, Zhang Ting, and Kang Suna. Fecal microbiota composition, their interactions, and metagenome function in us adults with type 2 diabetes according to enterotypes. International Journal of Molecular Sciences, 24(11), 2023. 37298483 10.3390/ijms24119533 PMC10253423

[127] Hu Bing, Das Promi, Lv Xianglin, Shi Meng, Aa Jiye, Wang Kun, Duan Liping, Gilbert Jack A., Nie Yong, and Wu Xiao-Lei. Effects of ‘healthy’ fecal microbiota transplantation against the deterioration of depression in fawn-hooded rats. mSystems, 7(3):e00218–22, 2022. 35481347 10.1128/msystems.00218-22 PMC9239139

[128] Wu Kaiwen, Luo Qiang, Liu Ye, Li Aoshuang, Xia Demeng, and Sun Xiaobin. Causal relationship between gut microbiota and gastrointestinal diseases: a mendelian randomization study. Journal of Translational Medicine, 22(1):92, 2024. 38263233 10.1186/s12967-024-04894-5 PMC10804519

[129] Paramsothy Sudarshan, Nielsen Shaun, Kamm Michael A., Deshpande Nandan P., Faith Jeremiah J., Clemente Jose C., Paramsothy Ramesh, Walsh Alissa J., van den Bogaerde Johan, Samuel Douglas, Leong Rupert W. L., Connor Susan, Ng Watson, Lin Enmoore, Borody Thomas J., Wilkins Marc R., Colombel Jean-Frederic, Mitchell Hazel M., and Kaakoush Nadeem O.. Specific bacteria and metabolites associated with response to fecal microbiota transplantation in patients with ulcerative colitis. Gastroenterology, 156(5):1440–1454.e2, 2025/10/30 2019. 30529583 10.1053/j.gastro.2018.12.001

[130] Xia Y., Wang J., Fang X., Dou T., Han L., and Yang C.. Combined analysis of metagenomic data revealed consistent changes of gut microbiome structure and function in inflammatory bowel disease. Journal of Applied Microbiology, 131(6):3018–3031, 12 2021. 34008889 10.1111/jam.15154

[131] Chierico Federica Del, Cardile Sabrina, Baldelli Valerio, Alterio Tommaso, Reddel Sofia, Bramuzzo Matteo, Knafelz Daniela, Lega Sara, Bracci Fiammetta, Torre Giuliano, Maggiore Giuseppe, and Putignani Lorenza. Characterization of the gut microbiota and mycobiota in italian pediatric patients with primary sclerosing cholangitis and ulcerative colitis. Inflammatory Bowel Diseases, 30(4): 529–537, 09 2023. 10.1093/ibd/izad203 PMC10988104 37696680

[132] Hochuli Nathaniel, Kadyan Saurabh, Park Gwoncheol, Patoine Cole, and Nagpal Ravinder. Pathways linking microbiota-gut-brain axis with neuroinflammatory mechanisms in alzheimer’s pathophysiology. Microbiome Research Reports, 3(1), 2024. 10.20517/mrr.2023.39 PMC10917618 38455083

[133] Ha Sung Min, Lee Kihyun, Kim Gun-Ha, Hurych Jakub, Cinek Ondřej, and Shim Jung Ok. Gutmicrobiota-based ensemble model predicts prognosis of pediatric inflammatory bowel disease. iScience, 27(12), 2025/10/30 2024. 10.1016/j.isci.2024.111442 PMC11650326 39691780

[134] Tsai Yu-Chieh, Tai Wei-Chen, Liang Chih-Ming, Wu Cheng-Kun, Tsai Ming-Chao, Hu Wan-Hsiang, Huang Pao-Yuan, Chen Chien-Hung, Kuo Yuan-Hung, Yao Chih-Chien, and Chuah Seng-Kee. Alternations of the gut microbiota and the firmicutes/bacteroidetes ratio after biologic treatment in inflammatory bowel disease. J Microbiol Immunol Infect, 58(1):62–69, Feb 2025. 39393964 10.1016/j.jmii.2024.09.006

[135] Rhodes Jonathan M. The role of escherichia coli in inflammatory bowel disease. Gut, 56(5):610–612, May 2007. 17440180 10.1136/gut.2006.111872 PMC1942130

[136] Dolatabadi Razie Kamali, Feizi Awat, Halaji Mehrdad, Fazeli Hossein, and Adibi Peyman. The prevalence of adherent-invasive escherichia coli and its association with inflammatory bowel diseases: A systematic review and meta-analysis. Frontiers in Medicine, Volume 8 – 2021, 2021. 10.3389/fmed.2021.730243 PMC8678049 34926490

[137] Martinez-Medina Margarita and Garcia-Gil Librado Jesus. Escherichia coli in chronic inflammatory bowel diseases: An update on adherent invasive escherichia coli pathogenicity. World J Gastrointest Pathophysiol, 5(3):213–227, Aug 2014. 25133024 10.4291/wjgp.v5.i3.213 PMC4133521

[138] Alahdal Hadil, Almuneef Ghaida, Alkhulaifi Manal Muhammed, Aldibasi Omar, Aljouie Abdulrahman, Alharbi Othman, Almohawes Zakiah Naser, Basingab Fatemah, and Rejili Mokhtar. Gut microbiota composition in patients with crohn’s disease in saudi arabia. PLoS One, 19(4):e0299749, 2024. 38656971 10.1371/journal.pone.0299749 PMC11042705

[139] Bang Won Yeong, Moon Jin Seok, Kim Hayoung, Lee Han Bin, Kim Donggyu, Shin Minhye, Jung Young Hoon, Shin Jongbeom, and Yang Jungwoo. Therapeutic modulation of the gut microbiome by supplementation with probiotics (sci microbiome mix) in adults with functional bowel disorders: A randomized, double-blind, placebo-controlled trial. Microorganisms, 13(10), 2025. 41156743 10.3390/microorganisms13102283 PMC12566429

[140] Schirmer Melanie, Garner Ashley, Vlamakis Hera, and Xavier Ramnik J.. Microbial genes and pathways in inflammatory bowel disease. Nature Reviews Microbiology, 17(8):497–511, 2019. 31249397 10.1038/s41579-019-0213-6 PMC6759048

[141] Hall Brantley, Levy Sophia, Dufault-Thompson Keith, Arp Gabriela, Zhong Aoshu, Ndjite Glory Minabou, Weiss Ashley, Braccia Domenick, Jenkins Conor, Grant Maggie R., Abeysinghe Stephenie, Yang Yiyan, Jermain Madison D., Wu Chih Hao, Ma Bing, and Jiang Xiaofang. Bilr is a gut microbial enzyme that reduces bilirubin to urobilinogen. Nature Microbiology, 9(1):173–184, 2024. 10.1038/s41564-023-01549-x PMC10769871 38172624

[142] Giebel Helge-Ansgar, Kalhoefer Daniela, Gahl-Janssen Renate, Choo Yoe-Jin, Lee Kiyoung, Cho JangCheon, Tindall Brian J., Rhiel Erhard, Beardsley Christine, Aydogmus Ömer O., Voget Sonja, Daniel Rolf, Simon Meinhard, and Brinkhoff Thorsten. Planktomarina temperata gen. nov., sp. nov., belonging to the globally distributed rca cluster of the marine roseobacter clade, isolated from the german wadden sea. International Journal of Systematic and Evolutionary Microbiology, 63:4207–4217, 2013. 23793856 10.1099/ijs.0.053249-0

[143] Li Ding, Wang Liping, Jiang Fan, Zeng Xiang, Xu Qinzeng, Zhang Xuelei, Zheng Qiang, and Shao Zongze. Unveiling the microbial diversity across the northern ninety east ridge in the indian ocean. Frontiers in Microbiology, Volume 15 – 2024, 2024. 10.3389/fmicb.2024.1436735 PMC11458393 39380675

[144] Dong Rongshu, Zhang Jie, Huan Hengfu, Bai Changjun, Chen Zhijian, and Liu Guodao. High salt tolerance of a bradyrhizobium strain and its promotion of the growth of stylosanthes guianensis. Int J Mol Sci, 18(8), Jul 2017. 10.3390/ijms18081625 PMC5578016 28788047

[145] Yue Haitao, Sun Shuwen, Wang Ruiqi, Ma Xiaoyun, Shen Shiwei, Luo Yiqian, Ma Xiaoli, Wu Ting, Li Shuang, Yang Zhengyang, and Gong Yuxi. Study on the mechanism of salt relief and growth promotion of enterobacter cloacae on cotton. BMC Plant Biology, 23(1):656, 2023. 38114925 10.1186/s12870-023-04641-w PMC10729352

[146] Anand Gautam, Shrivas Vijay Laxmi, Dubey Shubham, Bhattacharjee Annapurna, and Sharma Shilpi. Stress-buster enterobacter sp. alleviates salinity stress in cajanus cajan together with impacting its rhizospheric microbiome. South African Journal of Botany, 156:202–212, 2023.

[147] Xia Fei, Hao Haiping, Qi Ying, Bai Hongtong, Li Hui, Shi Zhenxia, and Shi Lei. Effect of salt stress on microbiome structure and diversity in chamomile (matricaria chamomilla l.) rhizosphere soil. Agronomy, 13(6), 2023.

[148] Balaban Metin, Moshiri Niema, Mai Uyen, Jia Xingfan, and Mirarab Siavash. TreeCluster: Clustering biological sequences using phylogenetic trees. PLOS ONE, 14(8):e0221068, August 2019. ISSN 1932-6203. doi: 10.1371/journal.pone.0221068. URL http://dx.plos.org/10.1371/journal.pone.0221068. 31437182 PMC6705769

[149] Rachtman Eleonora, Jiang Yueyu, and Mirarab Siavash. Machine Learning Enables Alignment-Free Distance Calculation and Phylogenetic Placement Using k -Mer Frequencies. Molecular Ecology Resources, 25(8):e70055, November 2025. ISSN 1755-098X, 1755-0998. doi: 10.1111/1755-0998.70055. URL https://onlinelibrary.wiley.com/doi/10.1111/1755-0998.70055. 41077897 PMC12550494

[150] Zhu Qiyun, Mai Uyen, Pfeiffer Wayne, Janssen Stefan, Asnicar Francesco, Sanders Jon G., Belda-Ferre Pedro, Al-Ghalith Gabriel A., Kopylova Evguenia, McDonald Daniel, Kosciolek Tomasz, Yin John B., Huang Shi, Salam Nimaichand, Jiao Jian-Yu, Wu Zijun, Xu Zhenjiang Z., Cantrell Kalen, Yang Yimeng, Sayyari Erfan, Rabiee Maryam, Morton James T., Podell Sheila, Knights Dan, Li Wen-Jun, Huttenhower Curtis, Segata Nicola, Smarr Larry, Mirarab Siavash, and Knight Rob. Phylogenomics of 10,575 genomes reveals evolutionary proximity between domains bacteria and archaea. Nature Communications, 10(1):5477, 2019. 10.1038/s41467-019-13443-4 PMC6889312 31792218

